# Identification and evaluation of small-molecule inhibitors against the dNTPase SAMHD1 via a comprehensive screening funnel

**DOI:** 10.1016/j.isci.2024.108907

**Published:** 2024-01-13

**Authors:** Si Min Zhang, Cynthia B.J. Paulin, Huazhang Shu, Miriam Yagüe-Capilla, Maurice Michel, Petra Marttila, Florian Ortis, Henri Colyn Bwanika, Christopher Dirks, Rajagopal Papagudi Venkatram, Elisée Wiita, Ann-Sofie Jemth, Ingrid Almlöf, Olga Loseva, Femke M. Hormann, Tobias Koolmeister, Erika Linde, Sun Lee, Sabin Llona-Minguez, Martin Haraldsson, Hanna Axelsson, Kia Strömberg, Evert J. Homan, Martin Scobie, Thomas Lundbäck, Thomas Helleday, Sean G. Rudd

**Affiliations:** 1 Science for Life Laboratory (SciLifeLab), Department of Oncology-Pathology, Karolinska Institutet, 171 65 Stockholm, Sweden; 2 Chemical Biology Consortium Sweden, Science for Life Laboratory (SciLifeLab), Department of Medical Biochemistry and Biophysics, Karolinska Institutet, 171 65 Stockholm, Sweden; 3 Weston Park Cancer Centre, Department of Oncology and Metabolism, University of Sheffield, Sheffield S10 2RX, UK

**Keywords:** Biochemistry, Molecular biology

## Abstract

SAMHD1 is a dNTP triphosphohydrolase governing nucleotide pool homeostasis and can detoxify chemotherapy metabolites controlling their clinical responses. To understand SAMHD1 biology and investigate the potential of targeting SAMHD1 as neoadjuvant to current chemotherapies, we set out to discover selective small-molecule inhibitors. Here, we report a discovery pipeline encompassing a biochemical screening campaign and a set of complementary biochemical, biophysical, and cell-based readouts for rigorous characterization of the screen output. The identified small molecules, TH6342 and analogs, accompanied by inactive control TH7126, demonstrated specific, low μM potency against both physiological and oncology-drug-derived substrates. By coupling kinetic studies with thermal shift assays, we reveal the inhibitory mechanism of TH6342 and analogs, which engage pre-tetrameric SAMHD1 and deter oligomerization and allosteric activation without occupying nucleotide-binding pockets. Altogether, our study diversifies inhibitory modes against SAMHD1, and the discovery pipeline reported herein represents a thorough framework for future SAMHD1 inhibitor development.

## Highlights

• Report a rigorous SAMHD1 chemical probe discovery pipeline
• Identified a series of low micromolar SAMHD1 inhibitors, exemplified by TH6342
• TH6342 deterred SAMHD1 dimerization *in vitro*, a prerequisite for catalysis

## Graphical abstract

**Figure:**
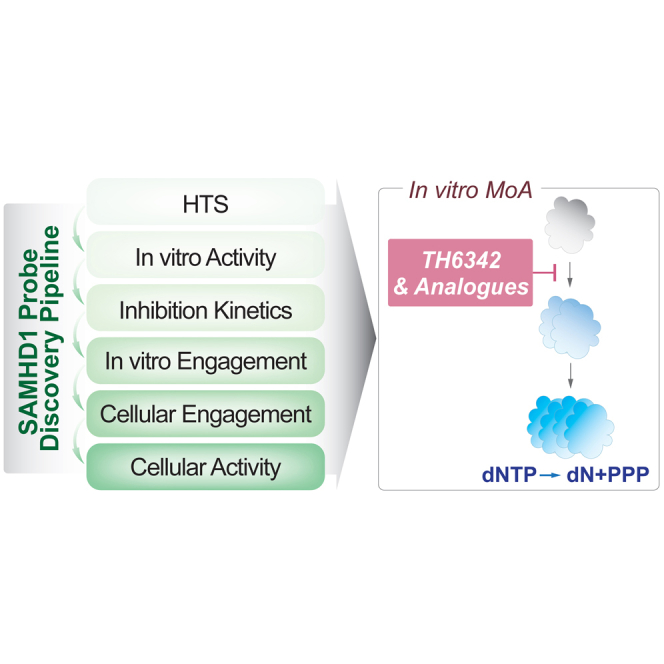

## Introduction

Sterile alpha motif and histidine-aspartic acid domain-containing protein-1 (SAMHD1) is a deoxynucleoside triphosphate (dNTP) triphosphohydrolase with critical roles in human health and disease.^1^^,^^2^ Belonging to the HD-domain superfamily, a group of metal-dependent phosphohydrolases,^3^ SAMHD1 catalyzes the hydrolysis of canonical dNTPs producing the cognate deoxynucleoside and inorganic triphosphate.^4^^,^^5^ This activity is regulated allosterically by sequential nucleotide binding, as each SAMHD1 monomer has two allosteric sites with distinct nucleotide-binding properties in addition to a catalytic site (reviewed in ref.^6^). Allosteric site 1 (AS1) binds to guanine nucleotides such as GTP or dGTP, which promotes formation of the SAMHD1 dimer, whereas allosteric site 2 (AS2) binds any canonical dNTP and promotes formation of the catalytically competent homotetramer.^7^^,^^8^^,^^9^ Given that dNTP hydrolysis by SAMHD1 is regulated by the abundance of (d)NTPs, the enzyme acts as a sensor and regulator of cellular nucleotide pool composition.^10^

Adding further to its biological function, SAMHD1 also plays a non-catalytic role in DNA repair, where it is responsible for recruiting enzymes to sites of damage or stalled DNA synthesis,^11^^,^^12^ and this role is linked to the ability of SAMHD1 to suppress the innate immune response.^11^^,^^13^ Accordingly, the diverse roles of this enzyme have several implications for human disease. Germline mutations in SAMHD1 are associated with the rare hereditary auto-inflammatory disorder Aicardi-Goutières syndrome^14^ together with early-onset stroke.^15^ SAMHD1 mutations are also found in many cancer types, including chronic lymphocytic leukemia (CLL),^16^^,^^17^ T cell prolymphocytic leukemia,^18^ colon cancer,^19^ and mantle cell lymphoma,^20^^,^^21^^,^^22^ among others (recently reviewed in ref.^23^).

SAMHD1 was also identified as a human immunodeficiency virus type-1 (HIV-1) restriction factor in myeloid cells^24^^,^^25^ and resting T cells.^26^ This is attributed to the ability of SAMHD1 to deplete dNTP pools below the level required for reverse transcription of the viral genome,^27^^,^^28^^,^^29^ although other functions of SAMHD1, such as nucleic acid binding, have also been shown to contribute.^30^ Demonstrating broad antiviral activity, SAMHD1 can also inhibit replication of other retroviruses^31^ and DNA viruses.^32^

In addition to viral restriction, given the dNTP hydrolase activity of SAMHD1 is critical for dNTP pool homeostasis,^10^ this has broad implications for cell fitness including maintaining the fidelity of genome duplication^33^ and efficient DNA repair^34^ including class-switch recombination.^35^ The dNTP hydrolase activity is also relevant for the activity of nucleobase and nucleoside analogs, a class of chemotherapy that is critical in the treatment of viral infections and cancer.^36^^,^^37^ These therapies are synthetic mimics of endogenous nucleobases and nucleosides and require bioactivation, typically sequential phosphorylation, inside target cells to exert their anti-viral or anti-cancer properties. The active species of several of these therapies are their triphosphate metabolite, and SAMHD1 is capable of hydrolyzing a number of these, thus converting them back to their inactive prodrug form.^38^^,^^39^^,^^40^^,^^41^^,^^42^^,^^43^^,^^44^ Accordingly, SAMHD1 can modulate the efficacy of several of these drugs in disease models.^39^^,^^40^^,^^41^^,^^42^^,^^43^^,^^44^^,^^45^^,^^46^^,^^47^^,^^48^^,^^49^ In the case of the deoxycytidine analog cytarabine (ara-C), the standard of care in acute myeloid leukemia (AML), it has been shown to dictate treatment efficacy in the clinic.^39^^,^^40^^,^^50^

To fully decipher the function(s) of SAMHD1 and their role in various biological processes and to investigate its therapeutic potential in nucleoside-based oncology treatment,^51^ SAMHD1-specific probes with different chemotypes/inhibitory mechanisms are clearly warranted. We and others have previously demonstrated that viral protein-X (Vpx), a simian/human immunodeficiency virus (e.g., SIV and HIV-2) accessory protein, can serve as a biological inhibitor, given its ability to target SAMHD1 for proteasomal degradation,^39^ albeit challenges remain for its delivery and target specificity.^45^ Furthermore, inhibitors of the key nucleotide biosynthetic enzyme ribonucleotide reductase (RNR) can be re-deployed to alter the intracellular nucleotide pool and thereby indirectly suppress the intracellular ara-CTP hydrolysis activity of SAMHD1 in various models of AML,^52^ which is now being evaluated in a clinical trial.^53^ Previous studies have also explored direct pharmacological inhibition of the dNTP hydrolase activity of SAMHD1,^38^^,^^41^^,^^54^^,^^55^^,^^56^^,^^57^^,^^58^ mainly centering around non-hydrolysable nucleoside triphosphates.^38^^,^^54^^,^^57^^,^^58^ Although the structural similarities to canonical dNTPs allow these molecules to target SAMHD1 in a competitive manner, and thereby broadened our knowledge of SAMHD1 enzymology, their triphosphate moieties prevent good cell permeability and hence limit applications to *in vitro* biochemical studies. Additional past efforts include focused biochemical screening campaigns with a selection of approved therapeutics.^55^^,^^56^ The identified drugs demonstrated apparent inhibitory activities *in vitro* with IC_50_ values in the 20–100 μM range, but no further studies of their mechanism of action, selectivities, or cell activities have been explored.^55^^,^^56^

To allow comprehensive SAMHD1 inhibitor characterization, and to identify potential alternative chemotypes, we established a SAMHD1 inhibitor screening funnel composed of complimentary biochemical, biophysical, and cellular assays, and here, we report the first dimerization deterring non-nucleotide inhibitors. Following the funnel, we first conducted a biochemical screening campaign of a diverse library of 17,656 small molecules, which, together with subsequent medicinal chemistry efforts, resulted in a collection of low μM inhibitors (TH6342, TH7127, and TH7528), with specificity for SAMHD1 versus other nucleotide-processing enzymes. We additionally identified a structurally related but inactive analog TH7126 that represents a suitable negative control. Subsequent mechanistic characterizations via enzymatic and target engagement assays revealed that TH6342 and analogs could deter dimerization of SAMHD1 and thereby its allosteric activation, representing a new mode of inhibition. The inhibitor characterization pipeline was further complemented with in-cell target engagement and SAMHD1 activity reporter assays. Although we show that the herein identified chemotype could not inhibit cellular SAMHD1 despite target engagement in cell lysates, TH6342 and analogs, together with competitive SAMHD1 inhibitors as well as viral Vpx protein, constitute a multifaceted set of tools in deciphering SAMHD1 enzymology and functions.

## Results

### Screening campaign for putative SAMHD1 inhibitors

**Figure 1:**
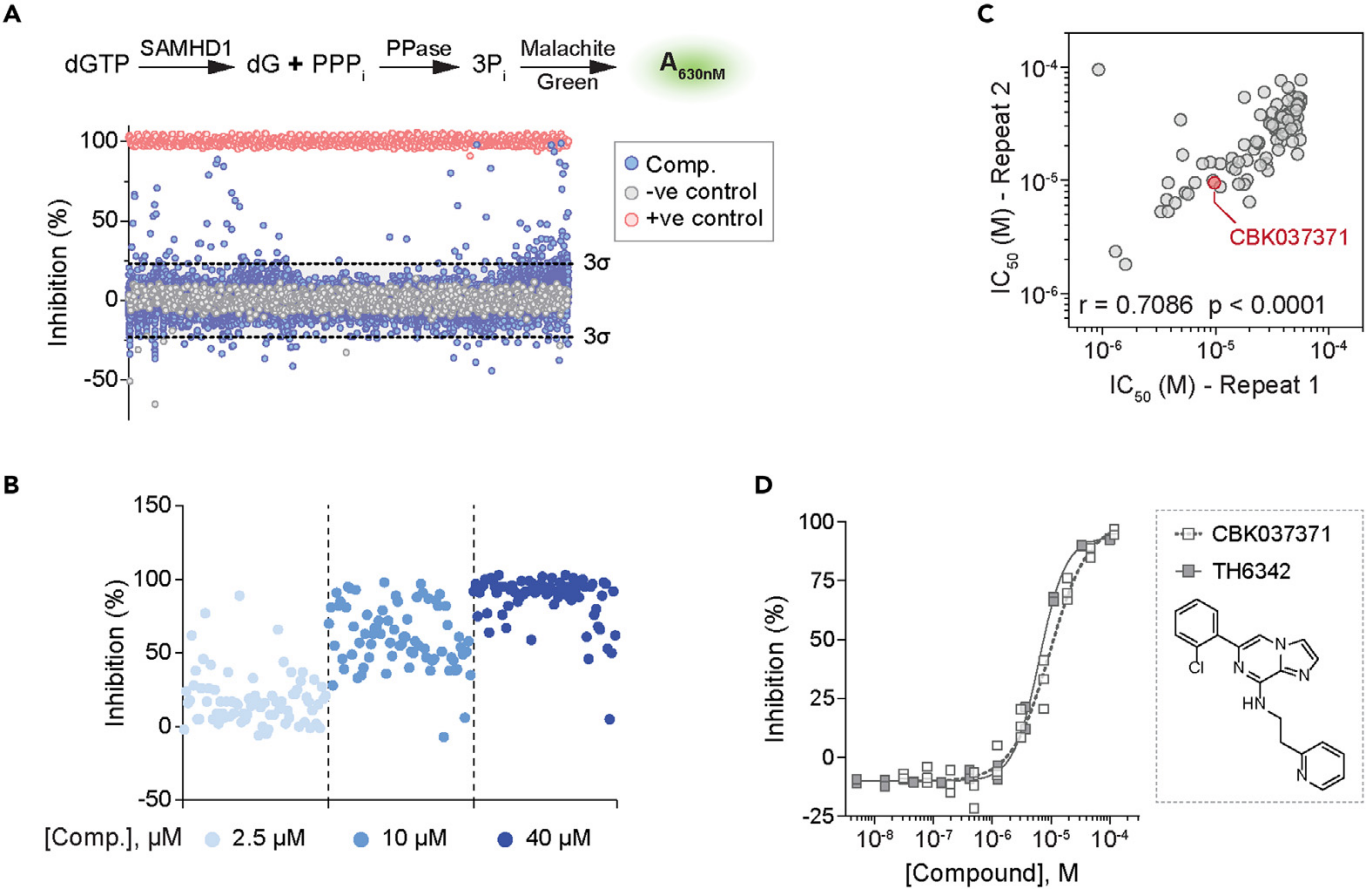
A high-throughput screening campaign to identify putative SAMHD1 inhibitors (A) High-throughput screen of 17,656 compounds using the enzyme-coupled malachite green (MG) assay. (Top panel) Schematic presentation of enzyme-coupled MG assay. In the assay, recombinant SAMHD1 was incubated with dGTP, and the reaction-released inorganic triphosphate (PPPi) was in turn broken down by inorganic pyrophosphatase (PPase) to inorganic phosphate (Pi), which, following addition of MG reagent, can be quantified by measuring absorbance at 630 nm. (Bottom panel) Scatterplot of SAMHD1 inhibition (%) in the presence of library compounds at 5 μM. Hit identification criteria (dashed line) were defined as three times the standard deviation beyond the average inhibition for the screening library. Reaction buffers containing SAMHD1 and dGTP only without screening compounds (100% activity) and SAMHD1-free with dGTP only (0% activity) conditions served as negative and positive controls for SAMHD1 inhibition, respectively. (B) Confirmation of 75 hit compounds from the screening campaign, exemplified by three-point (2.5, 10, and 40 μM) dose-response inhibitions of recombinant SAMHD1 in the enzyme-coupled MG assay. (C) Confirmation of 94 selected hit compounds and their analogs via two independent 11-point dose-response MG experiments. Concentrations required to inhibit 50% enzymatic activity (IC_50_) from the two experiments agreed, with Spearman correlation r and p values indicated. Compound CBK037371 was selected as the chemistry starting point for further inhibitor development and is highlighted in red. (D) In-house synthesized CBK037371 (referred as TH6342 hereinafter) demonstrated similar activity as CBK037371 from screening campaign, shown with 11-point dose-response MG experiments. Individual inhibition % of n = 2–3 independent experiments are shown. See also Figures S1, S2, and S14 and Tables S1 and S2.

As the first step to develop small-molecule SAMHD1 inhibitors, we screened a small molecule library for potential inhibitors, utilizing a previously established and validated enzyme-coupled malachite green (MG) assay (Figure 1A).^39^^,^^59^ SAMHD1 produces inorganic triphosphate from dNTP hydrolysis, and this reaction was coupled to that of a pyrophosphatase, which converts the inorganic triphosphate into individual inorganic monophosphates. These can be readily measured using the MG assay, which we used to indirectly determine SAMHD1 activity (Figure 1A). In the screening campaign, dGTP was chosen as the substrate due to its ability to fully activate SAMHD1 through occupying both the AS1 and AS2 sites (Figures S1A and S1B). Using this assay, we screened a library comprising 17,656 distinct chemical entities at a single concentration of 5 μM (conducted by Chemical Biology Consortium Sweden,^60^ see Table S1). Performance of the screening was deemed excellent with an average Z′ factor of 0.75 (Figure S1C). Based on a hit criterion of apparent inhibition over three times the standard deviation from the average inhibition of the screening library, 75 hit compounds were identified, yielding a hit rate of 0.42%.

Primary hit compounds were subjected to a round of hit confirmation experiments at multiple concentrations (Figure 1B) and further triaged based on their purity and promiscuity qualities (Figure S2A). The resulting 48 final hit compounds, together with a curated library of their in-house available analogs, 200 compounds in total, were then evaluated with an 11-point dose-response curve (DRC) test. A subset of 96 compounds with confirmed activity were again re-examined in a second 11-point DRC test. Excellent correlation was observed between the two rounds of DRC confirmation, thus validating the screen output (Figures 1C, S2B, and S2C). CBK037371, a hit compound identified from the analog expansion and close analog of primary screen hit CBK037439 (see Table S2 for full screen data), exhibited μM inhibitory potency (half maximal inhibitory concentrations, IC_50_ = 9.6 μM) and was further validated through in-house re-synthesis and purification (referred to hereafter as TH6342). This compound was selected for further characterization as a starting point for medicinal chemistry optimization (Figure 1D).

### TH6342 and analogs selectively inhibited SAMHD1

We next initiated a medicinal chemistry follow-up around the initial hit compound TH6342, where key chemical features critical for potency were identified by monitoring IC_50_ activities in the MG assay. The structure-activity relationship (SAR) studies (Figure S3) were initiated by altering the pyridyl-ethyl-amino part employing a range of primary and secondary amines. A number of similarly active 1,2-diamino or pyridyl-ethyl-amino compounds were generated and confirmed this necessary modification in TH6342. We then directed our attention toward the 2-chloro-phenyl substituent. Phenyl analog and heterocycle synthesis led to the development of two compounds with moderately improved activity, TH7127 (2-MeO) and TH7528 (2-thiophenyl), and an inactive control analog TH7126 (2-Amino). In an attempt to explore the core of the molecule, we further synthesized a diverse set of heterocycles with 1,2- and 1,3-disubstitution. Finally, matched pair analysis was performed to build confidence in the series.

**Figure 2:**
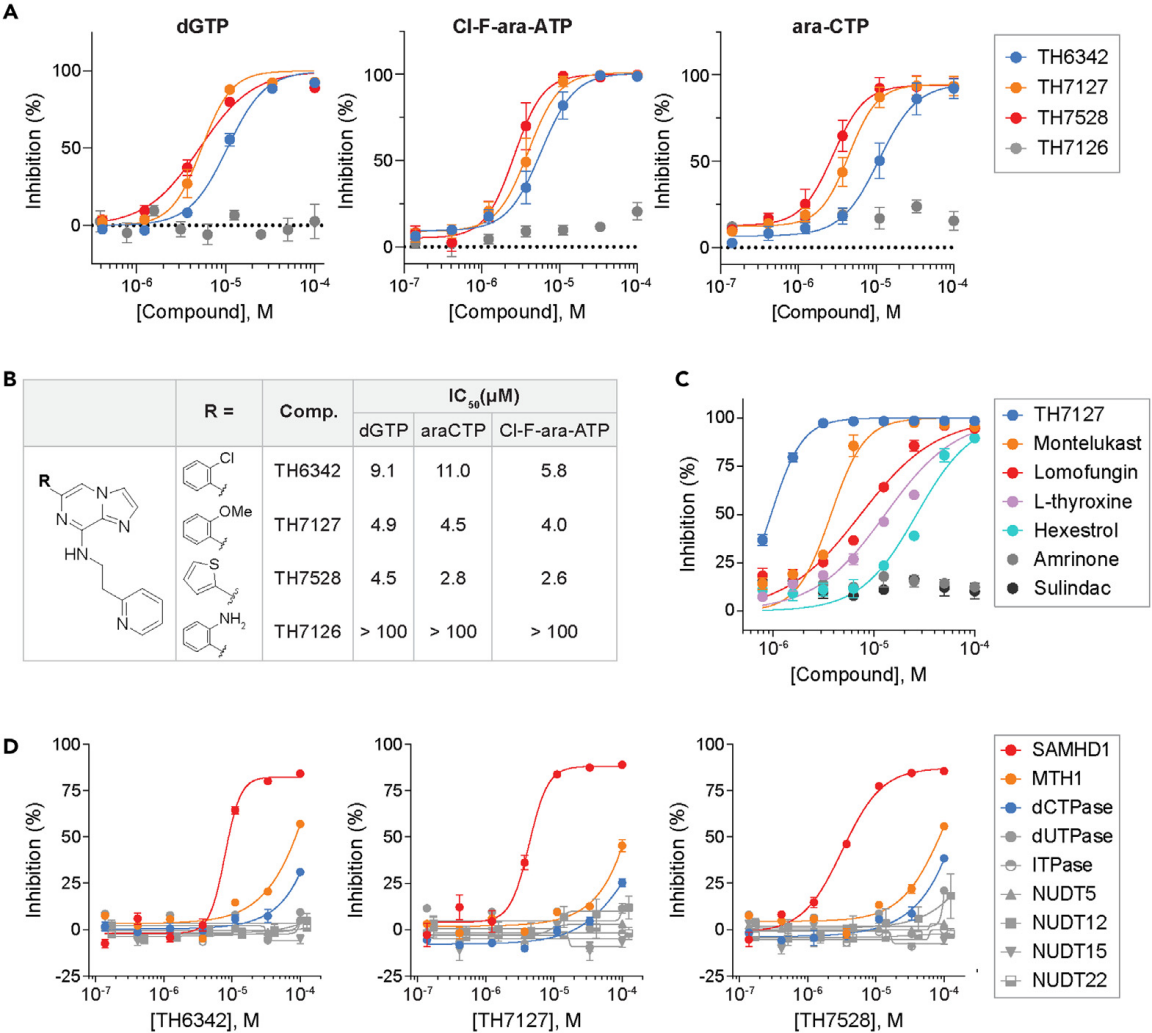
TH6342 and its analogs selectively inhibited SAMHD1 activity with low micromolar potency (A and B) TH6342, TH7127, and TH7528, but not TH7126, inhibited the enzymatic activities of SAMHD1 against dGTP, Cl-F-ara-ATP, and ara-CTP. In (A), mean inhibition % ± SEM of n = 3 independent experiments are shown. In (B), IC_50_ values were determined by curve-fitting mean inhibition % values from (A) using a nonlinear regression model (variable slope, GraphPad Prism). (C) TH7127 demonstrated superior biochemical potency compared with previously published SAMHD1 inhibitors. Mean inhibition % ± SEM of n = 2 independent experiments are shown. (D) TH6342, TH7127, and TH7528 maintained reasonable selectivity for SAMHD1, when assayed against a panel of nucleoside pyro-/tri-phosphatases. Mean inhibition % ± SEM of a representative experiment performed in sextuplicate are shown. Enzymatic activities of SAMHD1 in (A–D) were determined using enzyme-coupled MG assays. See also Figures S3 and S14 and Table S3.

Importantly, aside from the natural substrate dGTP, TH6342 as well as its active analogs TH7127 and TH7528 could also inhibit the hydrolysis of ara-CTP and Cl-F-ara-ATP, the active metabolites of the anti-leukemic drugs cytarabine and clofarabine, respectively. Under the same assay conditions, their close analog TH7126 conferred minimal inhibition, hence serving as a control compound for further mechanistic studies (Figures 2A and 2B). The low μM potency for TH7127 compared favorably against those of a panel of small-molecule non-nucleotide-based therapeutics previously reported to inhibit SAMHD1 *in vitro*^56^ (Figure 2C). Most critically, we were able to show that TH6342 and the analogs TH7127 and TH7528 selectively inhibited SAMHD1, when assayed up to 100 μM against a panel of nucleotide phosphatases of diverse substrate preferences (Figure 2D). Additionally, the assay systems for these enzymes utilized similar assay conditions such as the choice of coupled enzyme as well as signal detection methodology (Table S3), hence offsetting assay interference and further facilitating the selection of SAMHD1-specific inhibitors.

### TH6342 and analogs deterred recombinant SAMHD1 oligomerization

**Figure 3:**
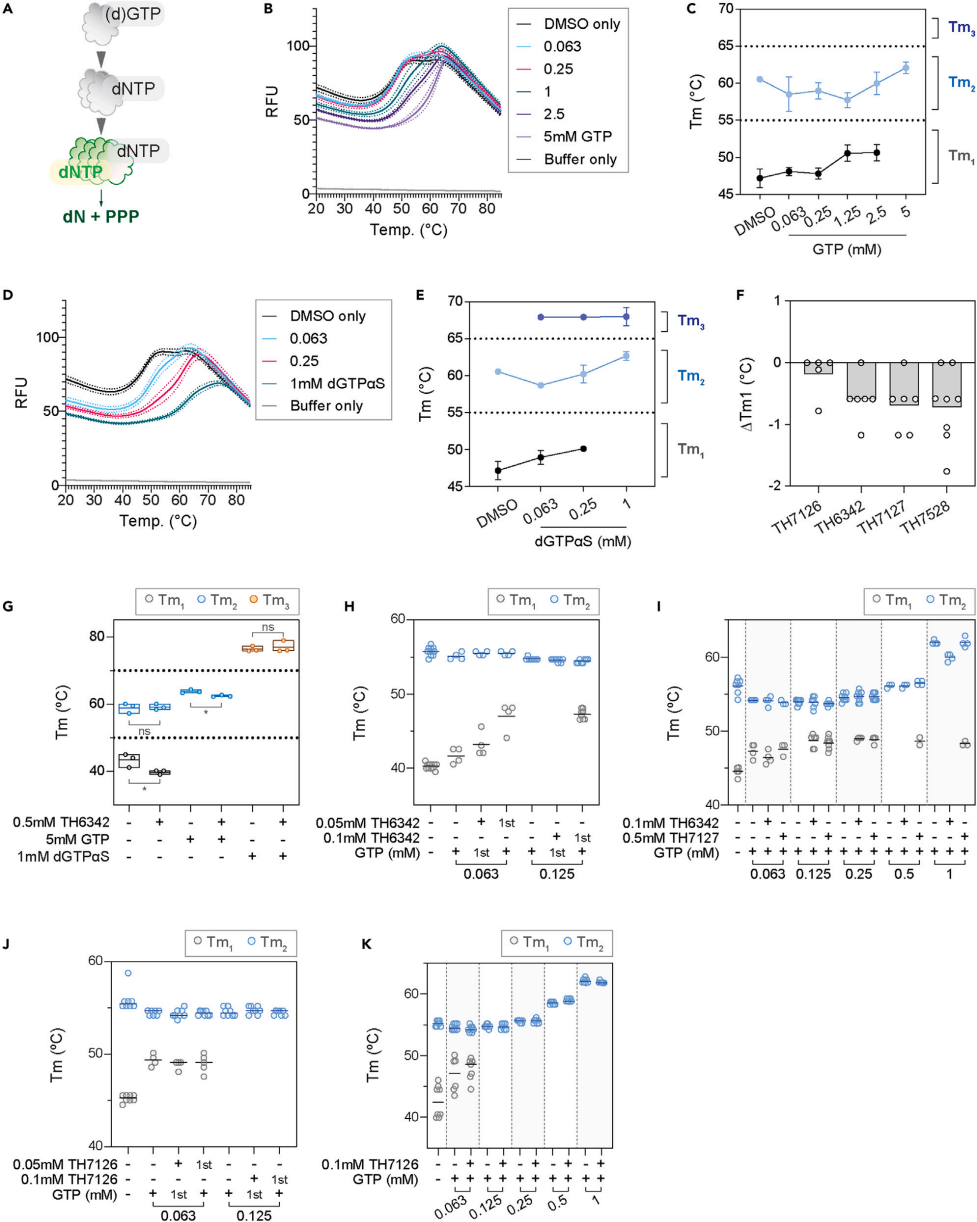
TH6342, TH7127, and TH7528 directly interacted with recombinant SAMHD1 and impeded GTP-induced dimerization (A**)** Recombinant SAMHD1 protein exhibited two apparent melting temperatures (Tm) in differential scanning fluorometry (DSF) experiments. Schematic representation of ordered activation of SAMHD1 – SAMHD1 becomes catalytically competent upon dimerization as induced by (d)GTP-binding to allosteric site 1 (AS1) and subsequently, tetramerization by dNTP-binding to allosteric site 2 (AS2). (B–E) Melting profile of recombinant SAMHD1 protein in the presence of GTP (B and C) or dGTPαS (D and E). Recombinant SAMHD1 protein was incubated with up to 5 mM GTP (B and C), 1 mM dGTPαS (D and E), or equal volume of DMSO, before its thermal stability being examined by DSF. Mean fluorescence signals (solid line) ± SEM (dashed line) of a representative experiment performed in quadruplicate are shown in (B) and (D). Melting temperatures (Tm) were determined as the minima of negative derivative of the melting curve, and mean Tm ± SD of n = 3–4 independent experiments each performed in quadruplicate are shown in (C) and (E). GTP and dGTPαS delayed the heat-induced denaturation of SAMHD1 and increased the Tm of SAMHD1 in a dose-dependent manner. (F) TH6342, TH7127, and TH7528, applied at 0.2–0.25 mM, effectively reduced the first apparent melting temperature (Tm_1_) of recombinant SAMHD1 in DSF experiment. Mean changes of Tm_1_ (ΔTm_1_) of n = 2–3 independent experiments performed in triplicates or quadruplicates are shown, where the dots represent individual values of technical repeats in each experiment. (G) TH6342 at 0.5 mM decreased the Tm of recombinant SAMHD1 protein in the presence of GTP. Mean SAMHD1 melting temperatures of n = 3 independent experiments are shown, together with the individual experiment values. Student’s t tests (unpaired, two-tailed) were performed across treatment groups [Tm_1_ (DMSO) vs. Tm_1_ (TH6342), p = 0.041, t ratio = 2.973, df = 4; Tm_2_ (DMSO) vs. Tm_2_ (TH6342), p = 0.84, t ratio = 0.2152, df = 4; Tm_2_ (GTP) vs. Tm_2_ (GTP+TH6342), p = 0.014, t ratio = 4.133, df = 4; Tm_3_ (dGTPαS) vs. Tm_3_ (dGTPαS + TH6342), p = 0.636, t ratio = 0.5116, df = 4], where asterisk signifies statistical significance (∗p ≤ 0.05, ∗∗p ≤ 0.01, ∗∗∗p ≤ 0.001, ∗∗∗∗p ≤ 0.0001). (H–K) Incubation with SAMHD1 inhibitors before, but not after GTP treatment deterred SAMHD1 dimerization. Recombinant SAMHD1 was subject to DSF assay after cotreatment with TH6342/TH7127/TH7126 and GTP. In (H) and (J), SAMHD1 was incubated with TH6342 (H) or TH7126 (J) and GTP of comparable concentrations in alternating orders, with compound added first labeled “1^st^.” In (I) and (K), SAMHD1 was incubated with 0.1 mM TH6342 or 0.5 mM TH7127 (I) or 0.1 mM TH7126 (K), prior to increasing concentrations of GTP. Mean SAMHD1 melting temperatures of n = 1–2 (H) or 2 (I–K) independent experiments are shown, together with the individual experiment values. See also Figures S4–S7 and S14.

The binding of TH6342, TH7127, and TH7528 to SAMHD1 *in vitro* was next interrogated using differential scanning fluorimetry (DSF), which evaluates ligand-binding based on target protein thermal stability. To achieve this, we first established assay conditions where we consistently observed stabilization by known ligands. In the absence of activating nucleotides, recombinant SAMHD1 mainly displayed two main melting temperatures (Tm), with the first one around 40°C–45°C (Tm_1_) and the second around 60°C (Tm_2_). SAMHD1 requires sequential nucleotide binding to become catalytically competent, with binding of (d)GTP to AS1 inducing dimerization and subsequent binding of a dNTP to AS2 inducing tetramer formation (Figure 3A). Addition of GTP to recombinant SAMHD1 (Figures 3B and 3C) led to a concentration-dependent transition away from the Tm_1_ at 40°C–45°C to Tm_2_ at 57°C–60°C, with the latter being the only observed species at 5 mM GTP. Despite variations in the content of folded apoprotein across individual experiments, the observed Tms are in good agreement with the known equilibrium between mono- and dimeric species for recombinant SAMHD1^61^ and with recently published thermal shift data for SAMHD1 by Orris et al*.*^62^ Although the latter study only quotes the first transition between 44°C and 46°C, the figures clearly show two transitions for SAMHD1 in the absence of ligands, which we interpret as representing monomeric (Tm_1_) and dimeric (Tm_2_) species, respectively. Importantly, although the observation of two transitions was not always observed for the apoprotein, signaling a less stable protein in absence of ligand, this was not the case in presence of nucleotides where we consistently observe ligand-induced stabilization.

Building on these observations, we next performed equivalent experiments replacing GTP with the non-hydrolysable analog dGTPαS, a known inducer of SAMHD1 tetramerization. As shown in Figures 3D and 3E, dGTPαS concentration dependently stabilized SAMHD1 beyond the first two transitions and elevated its melting temperature to a third Tm_3_ close to 70°C, which likely represents the tetrameric species. The observation of such significant stabilization is also in agreement with the study by Orris et al.*,*^62^ although they used elevated dGTPαS concentrations at 2 mM and observed a higher Tm_3_ of 74°C–76°C across different SAMHD1 forms. Our data clearly show how dGTPαS is a more potent stabilizer of SAMHD1, as the first (monomer) transition is largely lost already at 0.25 mM concentration.

Having established the Tm shift assay format for recombinant SAMHD1, we subsequently applied this to examine the interactions with inhibitors. In the absence of nucleotides, the inactive analog TH7126 did not alter SAMHD1 thermal stability at up to 200 μM concentration, whereas TH6342, TH7127, or TH7528 gave a small concentration-dependent reduction in Tm_1_ (Figures 3F and S4). These small changes clearly signaled that the inhibitors did not behave as the SAMHD1-stabilizing nucleotides, i.e., with a Tm shift toward the higher order species, raising the question whether these compounds inhibited SAMHD1 through irreversible inactivation and/or other mechanisms, e.g., interfering with SAMHD1 multimer stability or formation. To address this, TH6342 and analogs were next subjected to a series of order-of-addition experiments followed by the DSF assay.

At high concentrations of stabilizing nucleotide, with GTP at 5 mM or dGTPαS at 1 mM, which induce the formation of SAMHD1 dimers and tetramers, respectively,^61^ subsequent addition of 0.5 mM TH6342 mildly destabilized GTP-bound, but not dGTPαS-bound, SAMHD1 species (Figures 3G and S5A). Considering that SAMHD1 was challenged with a very high concentration of inhibitor compared with its inhibitory potency (∼50x IC_50_), we next coincubated SAMHD1 with TH6342 at only 5- and 10-fold above IC50 and reduced levels of GTP and furthermore at alternating orders. Under these conditions, the incubation with TH6342 following GTP did not significantly affect the protein melting profile (Figures 3H and S5B), signaling the inhibitor does not disrupt already established dimers. On the contrary, when SAMHD1 was first pre-incubated with 0.1 mM TH6342 or 0.5 mM TH7127, before the addition of a low concentration of GTP, the allosteric activator was not able to fully remove the Tm_1_ transition, suggesting the persistent presence of monomeric species (Figures 3H, 3I, and S5B–S5D). For 0.5 mM TH7127, this remained true also when increasing to higher GTP concentrations of 0.5–1 mM, where 0.1 mM TH6342 could not retain the monomeric species (Figures 3I, S5C, and S5D). As a control, the inactive analog TH7126 did not disrupt SAMHD1 dimerization, when added before or after GTP (Figures 3J, 3K, and S6). These observations were further supported by data from orthogonal assays determining SAMHD1 oligomerization based on protein sizes or direct visualization, i.e., dynamic light scattering (DLS) (Figures S7A and S7B) and *in vitro* chemical crosslinking (Figures S7C and S7D), respectively. Altogether, these data support a model in which TH6342 and analogs inhibited SAMHD1 by hampering GTP-induced dimerization and thereafter activation, though further kinetic studies were needed to decipher the mechanism of action.

### TH6342 and analogs impeded the allosteric activation of SAMHD1

**Figure 4:**
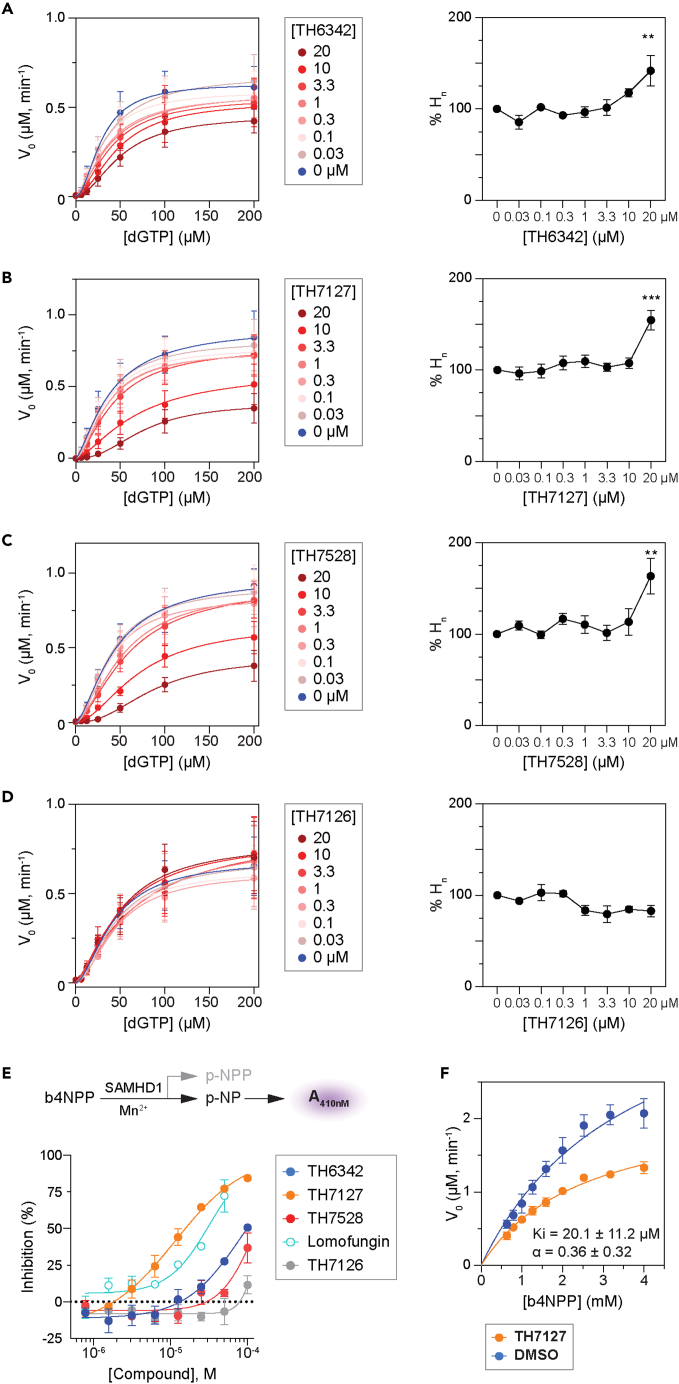
TH6342 and its analogs delayed the allosteric activation of SAMHD1 (A–D) Study of the inhibition mechanism using the enzyme-coupled MG assay, with dGTP as the substrate. (Left panels) Global fitting of the saturation curves using an allosteric sigmoidal model in GraphPad Prism. (Right panels) Fold changes of determined Hill coefficient values (% nH), compared with DMSO-treated group. Mean ± SEM of n = 3 independent experiments are shown. Ordinary one-way ANOVA (Dunnett’s multiple comparisons test) was performed between % nH in compound- versus DMSO-treated groups [for TH6342, % nH (0 μM) vs. % nH (0.03μM), p = 0.6445, q = 1.328, DF = 16; % nH (0 μM) vs. % nH (0.1μM), p = 0.9997, q = 0.1810, DF = 16; % nH (0 μM) vs. % nH (0.33 μM), p = 0.9802, q = 0.6349, DF = 16; % nH (0 μM) vs. % nH (1.1 μM), p = 0.9996, q = 0.3090, DF = 16; % nH (0 μM) vs. % nH (3.33 μM), p = 0.9998, q = 0.1265, DF = 16; % nH (0 μM) vs. % nH (10 μM), p = 0.4475, q = 1.632, DF = 16; % nH (0 μM) vs. % nH (20 μM), p = 0.0077, q = 3.862, DF = 16. For TH7127, % nH (0 μM) vs. % nH (0.03 μM), p = 0.9995, q = 0.3526, DF = 14; % nH (0 μM) vs. % nH (0.1 μM), p = 0.9999, q = 0.1210, DF = 14; % nH (0 μM) vs. % nH (0.33 μM), p = 0.9377, q = 0.8167, DF = 14; % nH (0 μM) vs. % nH (1.1 μM), p = 0.8539, q = 1.010, DF = 14; % nH (0 μM) vs. % nH (3.33 μM), p = 0.9995, q = 0.3290, DF = 14; % nH (0 μM) vs. % nH (10 μM), p = 0.9404, q = 0.8086, DF = 14; % nH (0 μM) vs. % nH (20 μM), p = 0.0003, q = 5.786, DF = 14. For TH7528, % nH (0 μM) vs. % nH (0.03μM), p = 0.9773, q = 0.6518, DF = 16; % nH (0 μM) vs. % nH (0.1μM), p > 0.9999, q = 0.0574, DF = 16; % nH (0 μM) vs. % nH (0.33 μM), p = 0.7561, q = 1.16, DF = 16; % nH (0 μM) vs. % nH (1.1 μM), p = 0.9639, q = 0.7160, DF = 16; % nH (0 μM) vs. % nH (3.33 μM), p = 0.9999, q = 0.0950, DF = 16; % nH (0 μM) vs. % nH (10 μM), p = 0.8864, q = 0.9309, DF = 16; % nH (0 μM) vs. % nH (20 μM), p = 0.0026, q = 4.389, DF = 16. For TH7126, % nH (0 μM) vs. % nH (0.03μM), p = 0.9568, q = 0.7441, DF = 16; % nH (0 μM) vs. % nH (0.1μM), p = 0.9994, q = 0.3647, DF = 16; % nH (0 μM) vs. % nH (0.33 μM), p = 0.9997, q = 0.2267, DF = 16; % nH (0 μM) vs. % nH (1.1 μM), p = 0.2579, q = 2.007, DF = 16; % nH (0 μM) vs. % nH (3.33 μM), p = 0.1080, q = 2.515, DF = 16; % nH (0 μM) vs. % nH (10 μM), p = 0.3206, q = 1.867, DF = 16; % nH (0 μM) vs. % nH (20 μM), p = 0.2194, q = 2.107, DF = 16.), where asterisk signifies statistical significance (∗p ≤ 0.05, ∗∗p ≤ 0.01, ∗∗∗p ≤ 0.001, ∗∗∗∗p ≤ 0.0001). (E) TH6342, TH7127, TH7528 inhibited SAMHD1 in a direct SAMHD1 enzymatic assay with B4NPP as the substrate. (Top panel) Schematic representation of the direct SAMHD1 enzymatic assay. In the presence of Mn^2+^ ions, SAMHD1 directly hydrolyses B4NPP into p-NPP and p-NP, with the latter being quantified by absorbance at 410 nm. (Bottom panel) TH6342, TH7127, and TH7528 inhibited the enzymatic activity of SAMHD1 against B4NPP, with superior activity compared with lomofungin. TH7126 exhibited minimal inhibition of SAMHD1. Mean inhibition % ± SEM of n = 2 independent experiments are shown and are further fitted with a non-linear regression model (dose-response, variable slope, four parameters, GraphPad Prism). (F) TH7127 inhibited SAMHD1 following the mixed inhibition model, determined using the direct SAMHD1 enzymatic assay as described in (C). Global fitting of the saturation curves, alone or in the presence of TH7127, supported the mixed inhibition model (GraphPad prism). Mean V_0_ ± SEM of n = 2 independent experiments are shown, with Ki and alpha values in the inset. See also Figures S8–S12 and S14.

To delineate the inhibition mechanisms by TH6342 and analogs, we conducted biochemical enzyme kinetic studies using the enzyme-coupled MG assay, with dGTP as the substrate. In agreement with previous studies,^54^^,^^61^ for reactions applying dGTP as both allosteric activator and substrate, the observed reaction kinetics displayed minimal cooperativity (Figure S8A). This can be explained by the relatively rapid allosteric activation to form long-lived SAMHD1 tetramers when compared with the timescale for subsequent steady-state substrate conversion. Interestingly, prior addition of SAMHD1 inhibitors, TH6342, TH7127, or TH7528, all dose-dependently impeded the dGTP-induced SAMHD1 activation, resulting in a shift from a hyperbolic to a sigmoidal dependence on substrate concentration, and significantly increased hill coefficients (H_n_). Meanwhile, the inactive analog TH7126 did not alter the reaction kinetics (Figures 4A–4D, S8B–S8E, and S9–S11).

Data from DSF and enzyme-coupled MG activity assays collectively suggested that the SAMHD1 inhibitors delayed the allosteric oligomerization of SAMHD1 and thereby its enzymatic activities. To circumvent the influences of allosteric sites and directly address if TH6342 and analogs could also act as competitive inhibitors by binding to the catalytic site, we employed a direct bis(4-nitrophenyl) phosphate (B4NPP) SAMHD1 activity assay. In the assay, sole presence of Mn^2+^ without nucleotides enables SAMHD1 to form catalytically competent active site that can accommodate and hydrolyze B4NPP.^56^ Processing of B4NPP results in the formation of yellow *p*-nitrophenol, thus allowing continuous kinetic measurements (Figures 4E, 4F, and S12A–S12C). Notably, the alternatively formed active site can also hydrolyze the canonical nucleotide substrates,^56^ suggesting no gross structural deviation between the active sites in the direct versus enzyme-coupled MG assays, hence allowing direct interrogation of the effect of TH6342 and analogs on the SAMHD1 catalytic pocket. Using this assay, we could confirm that TH6342, TH7127, and TH7528 retained their inhibitory activities (Figure 4E). Notably, TH7127 displayed higher activity than lomofungin, the most potent molecule previously identified using the B4NPP direct enzymatic assay. The inhibition mechanism of TH7127 was subsequently investigated using B4NPP as the substrate. Agreeing with inhibition mechanism suggested by enzyme-coupled MG assay, results from global fitting of the dataset from the B4NPP activity assay did not indicate direct competition for the catalytic pocket, but rather supported a mixed mode of inhibition (Ki = 20.1 μM, alpha = 0.36) (Figures 4F and S12D). Collectively, the data from kinetic studies, as corroborated by the DSF assay and orthogonal oligomerization assays, suggested that TH6342 and analogs inhibited SAMHD1 by delaying its allosteric activation.

### Interrogation of cellular activities of the SAMHD1 inhibitors

Past *in vitro* studies have identified effective SAMHD1 inhibitors including various nucleotide analogs^38^^,^^54^^,^^57^^,^^58^; still, cellular permeability and target engagement remain challenging, warranting the development of other chemical entities such as small molecule inhibitors. Having demonstrated the *in vitro* potency of TH6342 and analogs, we next interrogated their ability to engage and inhibit cellular SAMHD1.

**Figure 5:**
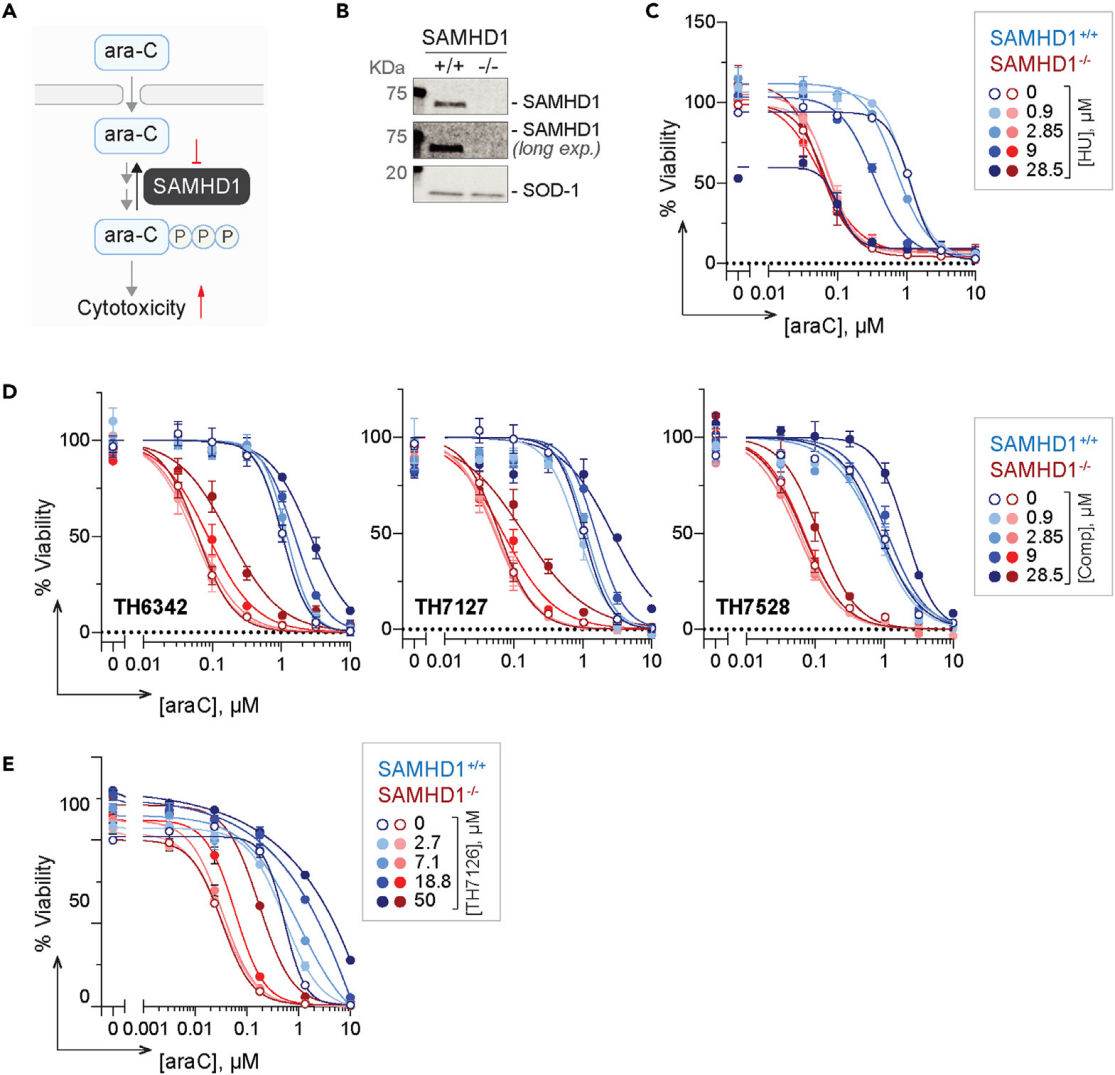
Indirect phenotypic readout to evaluate cellular SAMHD1 inhibition (A) Schematic representation of the phenotypic assay to indirectly assess cellular SAMHD1 inhibition. SAMHD1 hydrolyses triphosphorylated ara-C in cells and thereby limits its cytotoxicity, which is re-purposed here to indirectly assay the effects of putative SAMHD1 inhibitors on intracellular SAMHD1. Potential on-target inhibition of cellular SAMHD1 would translate into ara-C potentiation in a SAMHD1-dependent fashion, i.e., only in SAMHD1-competent (SAMHD1^+/+^) but not -deficient (SAMHD1^−/−^) cells. (B) SAMHD1 expression profile of SAMHD1 wild-type (SAMHD1^+/+^) and knockout (SAMHD1^−/−^) THP-1 cells. Western blots of a representative experiment are shown, with SOD-1 as the loading control. (C–E) SAMHD1^+/+^ and SAMHD1^−/−^ THP-1 cells were incubated with a dose-dependent concentration matrix of ara-C and hydroxyurea (HU) (C), SAMHD1 inhibitors (D), or negative control compound TH7126 (E) for four days, before cell viability was assessed by resazurin reduction assay. Resazurin signals were normalized to DMSO-treated control groups, and mean viability % ± SEM of n ≥ 2 independent experiments each performed in duplicates are shown.

SAMHD1 has been shown exhaustively to hydrolyze cytotoxic metabolites of nucleoside analogs such as cytarabine.^40^^,^^45^^,^^51^^,^^52^ Depletion of SAMHD1, or its indirect inactivation via RNR inhibitors (e.g., hydroxyurea), could lead to SAMHD1-dependent sensitization to these drugs,^39^^,^^40^^,^^52^ which in turn provides opportunities to evaluate cellular activities of putative SAMHD1 inhibitors (Figure 5A). Thus, we utilized a phenotypic assay in which THP-1 acute monocytic leukemia cells with SAMHD1 knockout or wild-type expression profile^39^ (Figure 5B) were incubated with a dose-response matrix of cytarabine and putative inhibitors for four days before cell viabilities were determined by resazurin reduction assay. Hydroxyurea, which was previously shown to induce SAMHD1-dependent cytarabine sensitization, served as the control (Figure 5C). Despite their *in vitro* potencies, none of TH6342, TH7127, or TH7528 sensitized THP-1 cells to cytarabine, regardless of the SAMHD1 expression status (Figure 5D). In contrast, hydroxyurea dose-dependently sensitized cells to cytarabine in a SAMHD1-dependent manner (Figure 5C). Notably, the inactive control analog TH7126, when challenged at 50 μM, antagonized cytarabine treatment, which is mildly observed for TH6342, TH7127, and TH7528 at around 30 μM as well, suggesting off-target effects of this series of chemotype at such high concentration tested (Figures 5D and 5E).

**Figure 6:**
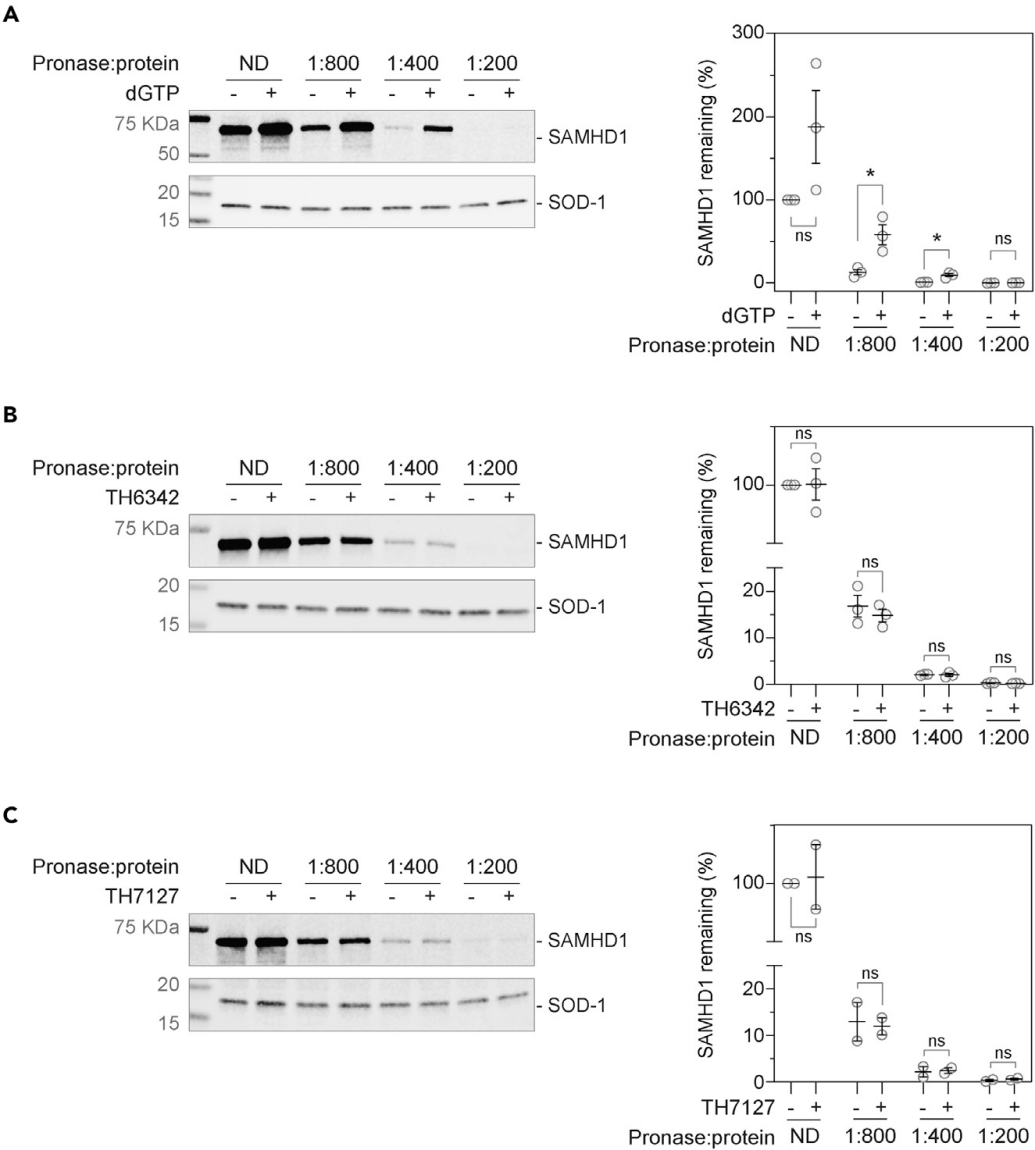
Evaluation of cellular engagement by the putative SAMHD1 inhibitors using the DARTS assay (A**)** Establishment of cellular SAMHD1 engagement DARTS assay using THP-1 cell lysate, with dGTP as the positive control. THP-1 cell lysates were incubated with 5 mM dGTP before being digested with pronase at indicated ratios to total protein concentration and then analyzed by western blot. (B and C**)** TH6342 (B) or TH7127 (C) did not show significant engagement of cellular SAMHD1 in THP-1 cell lysate in DARTS assays. THP-1 cell lysate was incubated with 100 μM compound or equivolume of DMSO, before being subject to pronase treatment at indicated enzyme/total protein ratio. Remaining soluble and folded proteins in the cell lysates were then analyzed by western blot. (A–C) (left panels) Representative western blot images. SOD-1 protein served as the loading control; (right panels) densitometry analysis, where SAMHD1 signals were normalized to SOD-1 signals and then relative to DMSO control samples received no digestion (ND). Mean relative protein signals ±SEM of n = 3 (A–B) or 2 (C) independent experiments are shown. In A–C, samples incubated with compounds were compared with DMSO control using Student’s t test (unpaired, two-tailed) [In (A), ND condition, p = 0.117, t ratio = 1.997, df = 4; pronase/protein ratio of 1:800, p = 0.022, t artio = 3.619, df = 4; pronase/protein ratio of 1:400, p = 0.010, t ratio = 4.570, df = 4; and pronase/protein ratio of 1:200, p = 0.091, t ratio = 2.219, df = 4. In (B), ND condition, p = 0.966, t ratio = 1.997, df = 4; pronase/protein ratio of 1:800, p = 0.500, t ratio = 0.740, df = 4; pronase/protein ratio of 1:400, p = 0.918, t ratio = 0.109, df = 4; and pronase/protein ratio of 1:200, p = 0.230, t ratio = 1.414, df = 4. In (C), ND condition, p = 0.859, t ratio = 0.201, df = 4; pronase/protein ratio of 1:800, p = 0.846, t ratio = 0.220, df = 4; pronase/protein ratio of 1:400, p = 0.864, t ratio = 0.193, df = 4; and pronase/protein ratio of 1:200, p = 0.400, t ratio = 1.061, df = 4.), and asterisk in figures signifies statistical significance (∗p ≤ 0.05, ∗∗p ≤ 0.01, ∗∗∗p ≤ 0.001, ∗∗∗∗p ≤ 0.0001). See also Figure S13.

**Figure 7:**
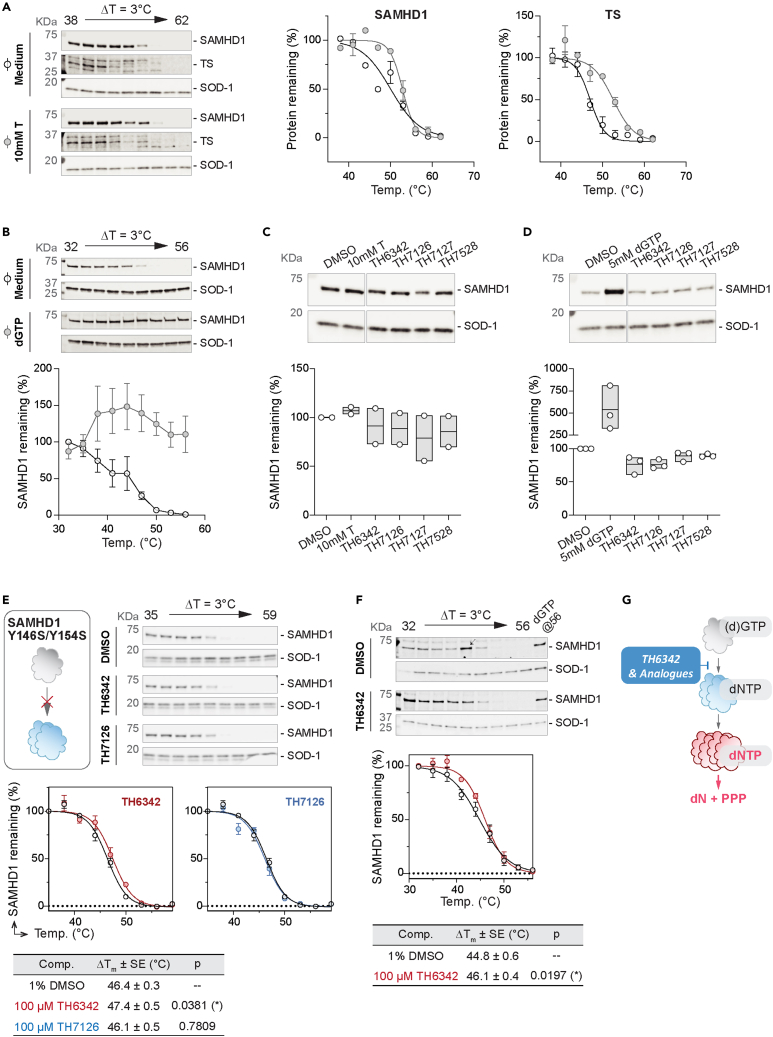
Evaluation of cellular engagement by the putative SAMHD1 inhibitors using CETSA (A and B) Establishment of cellular SAMHD1 engagement CETSA assay, validated by thymidine (A) or dGTP (B) as positive controls on intact THP-1 cells (A) or cell lysate (B), respectively. Intact THP-1 cells (A) or cell lysates (B) were incubated with 10 mM thymidine (A) or 5 mM dGTP (B), before being heated at indicated temperatures and analyzed by western blot. [Left (A) or top (B) panels] Representative western blot images where SOD-1 protein served as the loading control. [Right (A) or bottom (B) panels] Densitometry analysis, where SAMHD1 or thymidylate synthase (TS) signals were normalized to SOD-1 signals and then relative to DMSO control samples heated at the lowest temperatures. Mean relative SAMHD1 signal ±SEM of n = 2 independent experiments are shown. (C and D) Engagement of cellular SAMHD1 in intact THP-1 cells (C) or cell lysates (D), interrogated by isothermal single-dose fingerprint CETSA. Intact THP-1 cells (C) or THP-1 cell lysates (D) were incubated with 100 μM putative SAMHD1 inhibitors or positive control compounds [10 mM thymidine in (C) and 5 mM dGTP in (D)]. Following heating at screening temperatures, soluble SAMHD1 was examined via western blot. (Top panels) Representative western blot images where SOD-1 protein served as the loading control; (bottom panels) densitometry analysis, where SAMHD1 signals were normalized to SOD-1 signals and then relative to DMSO control samples heated at the lowest temperatures. Mean relative SAMHD1 signal of n = 2 (C) or 3 (D) independent experiments are shown with values of individual experiments. (E and F) TH6342, but not TH7126, mildly engaged cellular SAMHD1, shown with CETSA melting curves. Clarified lysates were prepared from THP-1 cells expressing wild-type SAMHD1 (F) or a dimerization-defective (Y146S/Y154S) mutant under knockout background (E). The lysates were then incubated with SAMHD1 inhibitors, 5 mM dGTP, or equivolume of DMSO, before being heated at indicated temperatures. Remaining soluble and folded proteins in the cell lysates were then analyzed by western blot. (Top panels) Representative western blot images where SOD-1 protein served as the loading control; arrow indicates the band excluded from densitometry analysis. (Middle panels) Densitometry analysis, where SAMHD1 signals were normalized to SOD-1 signals and then relative to DMSO control samples heated at the lowest temperatures. Mean relative SAMHD1 signal of n = 2 (E) or 4 (F) independent experiments are shown with SEM. (Bottom panels) Summary of SAMHD1 T_agg_ values, determined by curve-fitting SAMHD1 signals via a Boltzmann sigmoidal model (GraphPad Prism). Ordinary one-way ANOVA tests (E) or paired t test (F) were performed between SAMHD1 T_agg_ in compound- versus DMSO-treated groups (Figure 7E—T_agg_ (TH6342) vs. T_agg_ (DMSO), p = 0.0381, t = 3.550, DF = 3; T_agg_ (TH7126) Vs. T_agg_ (DMSO), p = 0.7809, t = 0.3042, DF = 3; Figure 7F—T_agg_ (TH6342) Vs. T_agg_ (DMSO), p = 0.0197, t = 4.567, df = 3.), where asterisk signifies statistical significance (∗ for p ≤ 0.05). (G) Proposed mechanism of action of TH6342 and analogs. We propose that the chemotypes identified herein, i.e., TH6342 and analogs, directly inhibited SAMHD1 hydrolase activities by deterring the enzyme dimerization, which is a prerequisite for formation of the catalytically competent SAMHD1 homotetramer. See also Figure S13.

Inadequate target engagement can often be attributed to poor cellular activities of small molecules, which was not explored in the previous studies of SAMHD1 inhibitors.^55^^,^^56^ In light of this, we therefore established a series of target engagement assays to examine ligand binding of SAMHD1 in a cellular context, specifically a drug affinity responsive target stability (DARTS) assay and a cellular thermal shift assay (CETSA) (Figures 6 and 7). DARTS measures on-target binding through monitoring resistance to protease (pronase) digestion, whereas for CETSA, thermal denaturation. Here, intact cells or cell lysates were incubated with potential ligands and then subjected to pronase treatment (DARTS) or heating (CETSA), before remaining folded SAMHD1 was detected via western blot. We could show that known interactors of SAMHD1, thymidine^62^ or dGTP, substantially stabilized SAMHD1 in intact cells or lysates, respectively, against heat- and/or pronase-induced denaturation, thus validating our assay setup (Figures 6A, 7A, and 7B).

We next screened the SAMHD1-binding potentials of TH6342 and its analogs via DARTS assay, where compound-treated cell lysates were subjected to increasing levels of pronase treatment. Neither TH6342 nor TH7127 in cell lysates altered SAMHD1 stability to pronase treatment (Figures 6B, 6C, and S13A). As an orthogonal approach, isothermal single-dose fingerprint CETSA^63^ was next conducted, where compound-treated cells or lysates were subjected to a single screening temperature (Figure S13B). Under the single-temperature setting, none of the tested inhibitors consistently altered the remaining SAMHD1 protein in the assay, either in intact cells or lysate (Figures 7C and 7D), indicating lack of engagement. This lack of engagement could be because the SAMHD1 inhibitors preferentially target monomeric SAMHD1, as indicated by biochemical studies, whereas cellular SAMHD1 is predisposed to multimerization due to intracellular GTP and dNTP pools. We next conducted CETSA using a SAMHD1^−/−^ THP-1 cell clone with rescue expression of SAMHD1^Y146S/Y154S^, a dimerization-defective SAMHD1 variant.^52^^,^^64^ Examining the full melt curves, we observed that TH6342, but not its inactive analog TH7126, mildly but significantly engaged SAMHD1^Y146S/Y154S^, increasing the aggregation temperature (T_agg_) by ∼1°C (Figure 7E). Similar data were observed using lysate from THP-1 cells expressing endogenous wild-type SAMHD1 (Figure 7F), suggesting an opportunity for this chemotype to target cellular SAMHD1. Altogether, TH6342 demonstrated mild target engagement in cell lysate; still, poor cell permeability and/or insufficient avidity toward SAMHD1 contributed to their low cellular activities, evidenced by the absence of synergy between the inhibitors and cytarabine. The data further highlighted the importance of cellular SAMHD1 engagement assays in characterizing intracellular potencies, supporting that it is an integral part of the screening funnel described here for the development of future potent and cell-active SAMHD1 inhibitors.

## Discussion

SAMHD1 has multifaceted roles important to human health and disease, through its enzymatic activity as a central dNTPase and through its non-catalytic activities.^2^ More recently, we and others have reported that SAMHD1 can also deactivate antileukemic drugs, notably cytarabine, the backbone therapy for AML, and thereby limit anticancer efficacy.^39^^,^^40^^,^^50^ To thoroughly decipher SAMHD1 biology and investigate its potential as an anticancer target, validated SAMHD1 probes that have undergone systematic and rigorous interrogations are warranted. Previous attempts have focused on screening FDA-approved libraries with limited hit characterization or dNTP analog inhibitors that mimic SAMHD1 substrates; however, their dNTP-like moieties disfavor cell permeability and/or high specificity. Here, in this study, we established a multidisciplinary SAMHD1-probe discovery pipeline and identified that a small molecule TH6342, and its analogs, inhibited SAMHD1 hydrolase activity *in vitro* with low μM potencies and high selectivity, and more interestingly, via a mode of inhibition that deters efficient allosteric activation of SAMHD1 without occupying (d)NTP binding pockets (Figure 7G).

We initiated this study with one of the largest reported biochemical screening campaigns against SAMHD1, composed of 17,656 diverse chemical entities of both commercial (Enamine, TimTec, Maybridge, and ChemDiv) and/or in-house origins (donation from Biovitrum). SAMHD1 requires subsequent occupancy of its two allosteric sites (AS1 and AS2) by (d)GTP and then any canonical dNTPs, respectively, to dimerize and eventually tetramerize into the catalytically competent species. The formed SAMHD1 tetramers further display impressive long half-life *in vitro*, making neither allosteric site accessible to free ligands,^61^ including potential inhibitors. Therefore, in the screening platform, recombinant SAMHD1 protein was incubated with the screening compounds prior to the addition of dGTP, the self-activating substrate, allowing the identification of both competitive inhibitors targeting the catalytic pockets, as well as compounds with alternative inhibitory mechanisms. During the revision of our work, a similar screening approach was reported^65^ but utilizing a higher dGTP concentration capable of saturating AS1 but not AS2, which was mixed with screening compounds prior to SAMHD1 addition. This approach would increase the likelihood of identifying competitive inhibitors at the catalytic site or AS2, but unfortunately, none of the screening hits were deemed promising for further development, highlighting that SAMHD1 is a challenging target.

From our screening campaign we subsequently identified and developed a collection of low-μM, direct, small-molecule SAMHD1 inhibitors, TH6342 and its close analogs TH7127 and TH7528, as well as their inactive analog TH7126 (Figure 2C). TH6342 and analogs further displayed an interesting mode of inhibition. Kinetic study using the MG enzyme-coupled assay with dGTP as the substrate demonstrated that they could dose-dependently increase the Hill coefficient of the reaction, suggesting delayed SAMHD1 activation (Figures 4A–4C). We further interrogated the effect of TH6342 and analogs on the catalytic pocket, through an orthogonal B4NPP direct enzymatic assay, where hydrolysis is initiated by Mn^2+^ without the canonical nucleotide-assisted ordered activation. We could confirm that TH6342 and analogs did not target the enzyme catalytic pocket, exemplified by the lack of competitive inhibition in this assay (Figure 4F). Instead, as corroborated by the order-of-addition experiment using DSF assay, pre-treatment of apoenzyme with TH6342 and analogs impeded GTP-induced dimerization, an essential step of SAMHD1 activation, further suggesting that the chemotypes reported herein delayed SAMHD1 activation by interrupting AS1 occupancy and/or SAMHD1 dimerization thereafter. Previous studies have developed several series of (deoxy)nucleotide-based SAMHD1 inhibitors for their structural mimicry to the canonical occupants of the allosteric/catalytic sites, such as dNMPNPP and dNTPαS that target the catalytic pocket and the enzyme-substrate complexes.^57^^,^^58^^,^^65^ Among them, the dUTP analog pppCH_2_dU inhibits SAMHD1 by delaying its activation, similar to the proposed mechanism of action for TH6342 and analogs (Figure 7G). Yet still, pppCH_2_dU demonstrated an apparent competitive nature of inhibition as it deters dimer-to-tetramer transition by predominately targeting AS2.^54^ We therefore envision that TH6342 and analogs, which specifically inhibited SAMHD1-mediated catalysis through deterring its dimerization, could complement the tetrameric SAMHD1-targeting dNTP analog inhibitors, as tool compounds not only to decipher SAMHD1 enzymology but also to potentially uncover new biological functions of SAMHD1 at different oligomeric states, e.g., as a nucleic-acid-binding protein in its monomeric state.^66^

Previous studies of SAMHD1 inhibitors focus on their utilities in *in vitro* studies with recombinant protein, inadequately addressing their behaviors in engaging and inhibiting cellular SAMHD1, partly due to the lack of appropriate assay systems. In this study, we further embarked on establishing a series of cellular target engagement assays and an indirect cellular SAMHD1 activity assay (Figures 5, 6, and 7). The cellular target engagement assays, i.e., CETSA and DARTS assays, assessing the on-target binding in both cell lysates and whole cells were positively validated by employing previously reported known binders to SAMHD1. We further took advantage of the well-established roles of SAMHD1 in hydrolyzing and thereby deactivating cytotoxic drugs (e.g., ara-CTP, the active metabolite of cytarabine) and re-purposed it as a proxy readout of the intracellular dNTPase activity of SAMHD1. Low SAMHD1 dNTPase activity, as indirectly elicited by hydroxyurea treatment,^52^ is reflected by dose-dependent sensitization with cytarabine-induced cytotoxicity (Figure 5C), in line with phenotypes of SAMHD1 abrogation via knockout^52^ or introduction of HIV-2 protein Vpx.^39^ The intracellular assay systems described here could therefore provide a structured way to evaluate intracellular behaviors of SAMHD1 inhibitors in both this work and future studies.

Being among the first SAMHD1 inhibitors examined for intracellular potencies, TH6342 showed evidence of engagement of SAMHD1 in THP1 cell lysates, though not when tested using whole cells. In line with this, TH6342 and analogs did not synergize with cytarabine, suggesting minimal inhibition of intracellular SAMHD1, despite inhibition of SAMHD1-mediated hydrolysis of ara-CTP *in vitro* (Figures 2A and 2B). While being the most potent direct SAMHD1 inhibitor of non-nucleotide moiety reported to date, the data indicated that TH6342 and analogs may require improvement in inhibitory potency and/or cell permeability. Nevertheless, cLog*P* values (TH6342, cLog*P* = 3.07; TH7127, cLog*P* = 2.31; and TH7528, cLog*P* = 2.24) predict favorable membrane permeability. One consideration is that high (d)GTP level in cells favors SAMHD1 dimerization, hence intrinsically hampering intracellular activities of monomer-targeting inhibitors, e.g., TH6342 and a recently reported dG-based AS1-occupying molecule.^65^ As the first step to interrogate this, here we established CETSA in THP-1 cells expressing a constitutively monomeric SAMHD1^Y146S/Y154S^ variant, in parallel with CETSA using wild-type cells. Interestingly, TH6342 significantly and equally engaged SAMHD1^Y146S/Y154S^ as well as wild-type SAMHD1 in THP-1 lysates. This indicates a window for inhibitors of similar mechanism-of-action to function in cells, awaiting to be validated with dedicated future studies. Additionally, SAMHD1 expression varies greatly among cells of different tissue lineages/origins,^10^^,^^52^ thus aside from THP-1 cells as a proof-of-concept model for compound screening, future compound evaluation could include additional cell lines/models, particularly those expressing a high SAMHD1 protein level and thereby presenting a large screening window.

In summary, starting with one of the largest screening campaigns against SAMHD1, here we identified and characterized a collection of SAMHD1 inhibitors, i.e., TH6342, TH7127, and TH7528, with low μM potency and high selectivity as underscored by an inactive analog TH7126. More intriguingly, these molecules displayed an allosteric mode of inhibition that deters efficient SAMHD1 activation. Although further improvement is needed for their intracellular potencies, we envision that TH6342 and analogs, together with SAMHD1 competitive inhibitors (e.g., dNTP analog compounds) as well as the SAMHD1-degradation-inducing viral Vpx protein, constitute a multifaceted set of tools in deciphering SAMHD1 enzymology and functions. Furthermore, this study established a comprehensive screening funnel encompassing biochemical, biophysical, and cell-based functional readouts, providing the community a thorough framework for future SAMHD1 inhibitor identification and development.

### Limitations of the study

In this study, we report a discovery pipeline for SAMHD1-targeting chemical probes encompassing a suite of biochemical, biophysical, and cell-based assays. To validate the applicability of each assay to assess the interaction of SAMHD1 (directly or indirectly) with a small molecule, we used known ligands (e.g., dGTP) or in the case of the phenotypic assay, a previously reported indirect pharmacological approach (i.e., RNR inhibitor). A validated cell-active small molecule inhibitor of SAMHD1 would be the ideal control to verify our assays, but at present no such probes have been reported, and thus we relied on the tools available. Given the oligomeric nature of SAMHD1, and its allosteric regulation, an ideal scenario would be to have multiple validated cell-active small molecule inhibitors, each with different modes of inhibition, to determine the applicability and limitations of each assay setup in relation to the mechanism of inhibition. Future studies can interrogate these points. A second output of this study is the report of an inhibitory chemotype of SAMHD1 (exemplified by TH6342) and establishing a new mode-of-inhibition, specifically deterring the dimerization step in the dNTP-induced oligomerization of this enzyme. The molecular determinants of this inhibition (e.g., ligand binding site) remain to be elucidated, and this limits our understanding of how these molecules function and hinders rational optimization of this chemotype. Future studies should also interrogate these points.

## STAR★Methods

### Key resources table

**Table:** 

REAGENT or RESOURCE	SOURCE	IDENTIFIER
**Antibodies**		

Mouse anti-SAMHD1 antibody	Abcam	OTI1A1; Cat# ab128107; RRID: AB_11142587
Rabbit anti-SAMHD1 antibody	Bethyl Laboratories Inc.	Cat# A303-691A; RRID: AB_11204405
Mouse anti-SOD-1	Santa Cruz Biotechnology, Inc.	G-11; Cat# sc-17767; RRID: AB_628301
Mouse anti-thymidylate synthase	Santa Cruz Biotechnology, Inc.	F-7; Cat# sc-376161; RRID: AB_10989925
Donkey anti-mouse IgG IRDye 800CW	Li-Cor	Cat# 926-32212; RRID: AB_621847
Donkey anti-rabbit IgG IRDye 800CW	Li-Cor	Cat# 926-32213; RRID: AB_621848
Donkey anti rabbit IgG IRDye 680RD	Li-Cor	Cat# 926-68073; RRID: AB_10954442
Donkey anti-mouse IgG IRDye 680RD	Li-Cor	Cat# 925–68072; RRID: AB_2814912

**Bacterial and virus strains**		

*E. coli* BL21(DE3)	Invitrogen	Cat# C600003

**Chemicals, peptides, and recombinant proteins**		

GTP	Sigma-Aldrich	Cat# G8877
Cytarabine	Sigma-Aldrich	Cat# C1768
Montelukast	Sigma-Aldrich	Cat# SML0101
L-thyroxine	Sigma-Aldrich	Cat# T2376
Hexestrol	Sigma-Aldrich	Cat# H7753
Sulindac	Sigma-Aldrich	Cat# S8139
Hydroxyurea	Sigma-Aldrich	Cat# H8627
Bis(4-nitrophenyl) phosphate	Sigma-Aldrich	Cat# N3002
4-Nitrophenol	Sigma-Aldrich	Cat# 1048
Sodium phosphate	Sigma-Aldrich	Cat# 342483
Thymidine	Sigma-Aldrich	Cat# T1895
Glutaraldehyde (25% aqueous solution)	Merck	Cat# 820603
Ara-CTP	Jena Bioscience	Cat# NU-1170
dGTPαS	Jena Bioscience	Cat# NU-424
Cl-F-ara-ATP	Jena Bioscience	Cat# NU-874
dGTP	GE Healthcare	Cat# 27-1870-04
Amrinone	Glentham Life Sciences	Cat# GP7331
Malachite green	Sigma-Aldrich	Cat# 213020; CAS:123333-61-9
His-tagged *E. coli* inorganic pyrophosphatase (PPase)	Generated in house using Protein Science Facility, Karolinska Institutet	N/A
SYPRO™ Orange Protein Gel Stain	Invitrogen	Cat# S6650
Pronase	Roche	Cat# 10165921001
Lomofungin	National Cancer Institute/Division of Cancer Treatment and Diagnosis/Developmental Therapeutics Program	NSC106995

**Deposited data**		

Source data for the Western blot images	This paper; Mendeley Data	https://doi.org/10.17632/8hmt8jbf7j.1

**Experimental models: Cell lines**		

Human: THP-1 ctrl	Herold et al.^39^; Rudd et al.^52^	N/A
Human: THP-1 g2c2	Herold et al.^39^; Rudd et al.^52^	N/A

**Recombinant DNA**		

pET28a(+) NUDT22	Carter et al.^67^	N/A
pET28a(+) dCTPase	Llona-Minguez et al.^68^	N/A
pET28a(+) dUTPase	Hagenkort et al.^69^	N/A
pET28a(+) MTH1	Svensson et al.^70^	N/A
pNIC28 NUDT5	SGC Stockholm	N/A
pNIC28 NUDT12	SGC Stockholm	N/A
pNIC28 NUDT15	SGC Stockholm	N/A
pNIC28 ITPase	SGC Stockholm	N/A
pET28a(+)	Novagen Merck Millipore	Cat# 69864-3
pET28a(+) SAMHD1	Herold et al.^1^	N/A

**Software and algorithms**		

GraphPad Prism 9	GraphPad	http://www.graphpad.com/scientific-software/prism/
Image Studio™ Lite Ver 5.2	LI-COR	https://www.licor.com/bio/image-studio-lite/download
ZS Xplorer software	Malvern Panalytical	https://www.malvernpanalytical.com/en/support/product-support/software/zetasizer-ultra-pro-zs-xplorer-software-update-v3-30
LightCycler® 480 Software	Roche	https://diagnostics.roche.com/global/en/products/instruments/lightcycler-480-ins-445.html#productSpecs

### Resource availability

#### Lead contact

Further information and requests for resources and reagents should be directed to and will be fulfilled by the lead contact, Sean Rudd (sean.rudd@scilifelab.se).

#### Materials availability

Materials and compounds generated in this study are available from the corresponding author on request.

#### Data and code availability

• Original western blot images have been deposited at Mendeley Data and are publicly available as of the date of publication. The DOI is listed in the key resources table.
• This paper does not report original code.
• Any additional information required to reanalyze the data reported in this paper is available from the lead contact upon request.

### Experimental model and study participant details

#### Cell lines

THP-1 (male) cells, i.e. THP-1 ctrl, and THP-1 g2c2 cells, were cultured in Iscove′s Modified Dulbecco′s Medium (IMDM) supplemented with 10% heat-inactivated fetal bovine serum (FBS) and penicillin/streptomycin (100 U/mL and 100μg/mL, respectively), at 37°C with 5% CO_2_ in a humidified incubator. The culture medium was purchased from ThermoFisher Scientific. THP-1 cells were purchased from ATCC, and its CRISPR-Cas9 derivative THP-1 ctrl (SAMHD1^+/+^) and THP-1 g2c2 (SAMHD1^-/-^) were generated and characterized as described previously.^39^^,^^52^ The cell lines were regularly monitored and tested negative for the presence of mycoplasma using a commercial biochemical test (MycoAlert, Lonza).

#### Microbe strain

E. coli BL21(DE3) was obtained from Invitrogen.

### Method details

#### Recombinant protein production & purification

Recombinant human SAMHD1 was expressed and purified as described before.^39^ Briefly, SAMHD1 was expressed from pET28a(+) (Novagen) vector with an N-terminal His-tag in *E.coli* BL21 (DE3) cells. It was then purified first using HisTrap HP (GE Healthcare) followed by SP cation-exchange (GE Healthcare) or HiLoad 16/60 Superdex 200 (GE Healthcare) columns (See Figure S14 for protein purity). Recombinant human MTH1, dCTPase, dUTPase, ITPase, NUDT5, NUDT12, NUDT15 and NUDT22 were expressed and purified as described previously.^71^^,^^67^^,^^72^^,^^73^^,^^68^^,^^69^^,^^70^ Briefly, all proteins were expressed with an N-terminal His-tag in *E.coli* BL21 (DE3) cells, where MTH1, dCTPase, dUTPase, NUDT22 were expressed from pET28a(+) (Novagen) vectors, and ITPase, NUDT5, NUDT12 and NUDT15 were expressed from pNIC28 vectors. All proteins, except dCTPase and dUTPase, were purified first using HisTrap HP (GE Healthcare) followed by gel filtration using HILoad 16/60 Superdex 75 (GE Healthcare). dCTPase and dUTPase were first purified using HisTrap HP (GE Healthcare), followed by His tag removal by thrombin (dUTPase) or TEV digestion (dCTPase), and were subsequently further purified using MonoQ ion exchange column.

All proteins were stored at -80°C in storage buffer (20 mM HEPES pH 7.5, 300 mM NaCl, 10% glycerol (v/v) and 0.5 mM TCEP) until further analysis. The Protein Science Facility (Karolinska Institutet, Sweden) performed expression and purification of all proteins, except for dCTPase and dUTPase.

#### Small molecule screening campaign

##### Small molecule library composition

The screening campaign comprised a combination of in-house and commercially available libraries, to a total of 17,656 compounds. The commercial compounds originate from Enamine, whereas the in-house libraries were partly donated by Biovitrum AB (the origin and composition have been described previously^74^). Compounds included in the screening set were selected to represent a diverse selection of a larger set of 65,000 compounds, while keeping a certain depth to allow crude SAR studies. The selection was also biased towards lead-like and drug-like profiles with regards to molecular weight, hydrogen-bond donors/acceptors and logarithm of partition coefficient (log P). For long-term storage, the compounds were kept frozen at −20°C as 10 mM solutions in DMSO under low-humidity conditions in REMP 96 Storage Tube Racks in a REMP Small-Size Store.

##### Small-molecule SAMHD1 inhibitor screening campaign

The screen for SAMHD1 inhibitors was conducted at Chemical Biology Consortium Sweden (www.cbcs.se), using the enzyme-coupled malachite green assay. Recombinant human SAMHD1 (0.26 μM) was incubated with 25 μM dGTP and *E. coli* PPase (12.5 U/mL), alone or in combination with screening compounds, in assay buffer (25 mM Tris-acetate at pH 8.0, 40 mM NaCl, 1 mM MgCl_2_, 0.005% Tween 20 and 0.3 mM TCEP) at room temperature (RT) for 20 minutes. Termination of the enzymatic reaction was done with 4 mM EDTA. The hydrolysis reaction was then measured by absorbance at 630 nm (read time of 0.1 s per well, Victor 3 from Perkin Elmer) after incubating with malachite green reagent for a minimum of 8 min under agitation.

To facilitate screening, aliquots of the compound stock solutions (10 mM in DMSO) were transferred to Labcyte 384 LDV plates (LP-0200) to enable dispensing using an Echo 550 acoustic liquid handler (LabCyte). Compound solutions were then dispensed at 10 nl/well directly into columns 1–22 of 384-well assay plates (Nunc 242757), while columns 23 and 24 were reserved for controls (see below). The plates were sealed with a peelable aluminium seal (Agilent 24210–001) using a PlateLoc thermal microplate sealer (Agilent) and kept at -20°C until used. The screening assay was conducted in a total assay volume of 20 μl per well in 384-well assay plates. The final compound concentration in the screen was 5 μM with a final DMSO concentration of 0.05% in all wells. On the day of screening, enzyme solution (10 μl/well) containing SAMHD1 and *E. coli* PPase and dGTP solution (10 μL/well) were added using a FlexDrop IV (Perkin Elmer) to assay plates already containing 10 nL of compound solutions. Following 20 minutes incubation at RT, 20 μL EDTA was added using a multidrop 384 (Thermo).

On each assay plate, column 24 contained dGTP and SAMHD1-free reaction buffer, and served as positive control (0% enzyme activity), while column 23 contained dGTP and SAMHD1 in reaction buffer but no compound and served as negative control (100% activity). Raw absorbance values at 630 nm were then normalised to negative and positive controls on each individual plate. Hit-limit was identified by the average plus three standard deviations of the library compound responses. Subsequent three-dose (2.5, 10 and 40 μM) and 11-point concentration response curves (ranging from 119 μM to 12.5 nM) was conducted using the same assay conditions.

#### Enzyme-coupled malachite green assays

##### Determination of SAMHD1 *in vitro* activity

Enzyme-coupled malachite green assays were used to determine the *in vitro* enzymatic activity of recombinant SAMHD1, performed as previously described.^39^^,^^59^ Briefly, 0.35 μM recombinant SAMHD1 was incubated with 25 μM dGTP in the reaction buffer (25 mM Tris-acetate pH 8, 40 mM NaCl, 1 mM MgCl_2_, 0.005% Tween-20, 0.3 mM TCEP) fortified with 12.5 U/mL *E. coli* PPase, in the presence or absence of compound treatment, at RT for 20 min. The reaction was then quenched by 7.9 mM EDTA (final concentration 3.95 mM), followed by addition of malachite green reagent (final concentration - 0.5 mM malachite green, 2.58 mM ammonium molybdate, 0.036% Tween-20) and incubation at RT for 20 min. Subsequently, absorption at 630 nm wavelength (A_630nm_) was acquired using a Hidex Sense microplate reader. For experiments using chemotherapeutic active metabolites as substrates, ara-CTP (200 μM) was tested with dGTPαS (3.125 μM) as AS1 and AS2 activator, and Cl-F-ara-ATP (50 μM) was tested with GTP (12.5 μM) as AS1 activator.

##### Study of compound selectivity against SAMHD1

Compound selectivity against recombinant MTH1, dCTPase, dUTPase, ITPase, NUDT5, NUDT12, NUDT15 and NUDT22 were evaluated using the enzyme-coupled malachite green assay as described before.^73^ Briefly, recombinant proteins were incubated with corresponding substrates in the reaction buffer (MTH1, NUDT5, NUDT12, NUDT15, dUTPase, and NUDT22 – 100 mM Tris-acetate pH 8, 40 mM NaCl, 10 mM Mg acetate, 0.005% Tween-20 and 1 mM DTT; ITPase – 100 mM Tris-acetate pH 8, 50 mM Mg acetate, 0.005% Tween-20 and 1 mM DTT; dCTPase – 100 mM Tris-acetate pH 8, 100 mM KCl, 10 mM MgCl_2_, 0.005% Tween-20 and 1mM DTT) fortified with corresponding phosphatases, in the presence or absence of compound treatment, for 20-40 min at RT. Malachite green reagent was then added. Following 15-20 min incubation at RT, A_630nm_ was measured. See Table S3 for specific assay conditions.

##### Kinetic study of TH6342 and analogues

Kinetic study of TH6342 and analogues was performed with the enzyme-coupled malachite green assay as described above, but with a dose-response concentration matrix of inhibitors (0.03-20 μM) and dGTP (6.25-200 μM). The reactions are performed on 384-well plates with a final volume of 20 μl and DMSO level controlled at 1%, where all reagents are dispensed using a D300e Digital Dispenser (Tecan). Specifically, the inhibitors and DMSO were dispensed first, followed by 5 μl reaction buffer, 5 μl reaction buffer containing recombinant SAMHD1 (final conc. 0.35 μM) and E. coli PPase (final conc. 12.5 U/mL), and finally 10 μl dGTP in reaction buffer. Following 20 min incubation at RT, the reaction was then quenched by EDTA, Subsequently, the malachite green reagent was added at 10 μl/well, and upon incubation at RT for 20 min, A_630nm_ was read in a Hidex Sense microplate reader. Standard curves of inorganic sodium phosphate were constructed to determine amount of phosphate released, and subsequently used to determine reaction kinetics (GraphPad Prism).

#### Dynamic light scattering (DLS)

SAMHD1 particle size distribution was determined using a Malvern ZETASIZER (Ultra) (Malvern Instruments). Specifically, samples were prepared by incubating recombinant SAMHD1 (2.8 - 9.1 mg/ml) with 1 mM GTP, 1 mM TH6342, or equivolume of DMSO (2%) in DLS buffer (25 mM Tris-acetate (pH = 7.5), 40 mM NaCl, 5 mM Mg acetate, 0.3 mM TCEP, 3% glycerol). Raw intensity-weighted data were converted to volume distribution by built-in algorithms to mitigate the impact of aggregated protein species.

#### *In vitro* chemical crosslinking

Recombinant SAMHD1 (10.4 μg) was incubated with TH6342 or equivolume of DMSO (2%) for 10 min at RT, before incubation with GTP and/or 1.875 mM glutaraldehyde for another 10 min at RT, all done in crosslinking buffer (20 mM HEPES (pH = 7.5), 40 mM NaCl, 5 mM MgCl_2_, 0.3 mM TCEP, 3% glycerol). The crosslinking reaction was terminated by 1 M Tris-HCl (pH = 7.5) in the same volume as glutaraldehyde. Protein multimers were then separated by SDS-PAGE, followed by Western blot.

#### B4NPP direct SAMHD1 enzymatic assay

B4NPP direct SAMHD1 enzymatic assay was performed as described.^56^ Briefly, 0.5 μM recombinant SAMHD1 was incubated with compounds or equivolume of DMSO control in the reaction buffer (50 mM Tris-acetate pH 8, 100 mM NaCl, 5 mM MnCl_2_, 0.5% DMSO, 2% glycerol, 0.5 mM TCEP) at RT for 10 min, following which 2 mM B4NPP was added and the reaction was then monitored *via* A_410nm_ using a Hidex Sense microplate reader. Reaction linearity was established by varying concentrations of SAMHD1 or B4NPP, in the absence of compound treatment. P-NP standard curve was established by measuring A_410nm_ of increasing concentrations of p-NP. Kinetic parameters of B4NPP hydrolysis by SAMHD1, in the absence or presence of compound treatment, were determined by globally fitting of the entire dataset using a mixed mode of inhibition (GraphPad Prism).

#### Target engagement assays

##### Differential scanning fluorometry (DSF)

DSF assay on recombinant SAMHD1 was conducted as described previously, with minor modifications.^73^ Briefly, 5 μM SAMHD1 in the assay buffer (25 mM Tris-acetate pH 7.5, 40 mM NaCl, 1 mM Mg acetate, 0.5 mM TCEP) fortified with Sypro Orange (5X, Invitrogen), at the final volume of 20 μL/well. The assay mixture was then subject to a 20-85/90°C temperature gradient for 20 min, with the fluorescence intensities (RFU) measured every second using a LightCycler 480 Instrument II (Roche Life Science). To assay the effects of compound and/or nucleotide treatments on SAMHD1 thermal stability, SAMHD1 was incubated with compounds or nucleotide for 10-20 min before data collection. When both nucleotides and compounds are present, SAMHD1 was incubated with nucleotides first for 10 min, and subsequently with compounds or equivolume of DMSO for 15 min before data collection. Melting temperatures (Tm) were identified by the minima of the negative first derivative (-*d*RFU/*d*T) of fluorescence intensity (LightCycler 480 Software).

##### Cellular thermal shift assay (CETSA)

For isothermal single-dose fingerprint CETSA using intact cells, THP-1 cells were incubated with 10 mM thymidine, 100 μM compounds, or equivolume of DMSO (1%) at 37°C and 5% CO_2_ in a humidified incubator. Two hours post-treatment, cells were collected, washed twice by PBS, and then resuspended in PBS supplemented with cOmplete Mini EDTA-free protease inhibitor cocktail (Roche, Merck) at 20 μL per 10^6^ cells, before the cells were heated at 53°C for 3 min in a Veriti 96-well Thermal Cycler (ABI). Samples were subsequently rested at RT for 3 min, supplemented with 40 μL sample buffer (50 mM HEPES pH 7.5, 5 mM β-glycerophosphate, 0.1 mM Na_3_VO_4_, 10 mM MgCl_2_, 2 mM TCEP, 1X cOmplete, Mini, EDTA-free protease inhibitor cocktail), and then lysed through three freeze-thaw cycles with alternating ethanol/dry ice and 37°C water bath. Finally, lysates were clarified by centrifugation at 17,000g for 20 min and then analysed by Western blot. For CETSA using cell lysates, THP-1 cells were first lysed in the sample buffer at 60 μL per 10^6^ cells, through three freeze-thaw cycles with alternating ethanol/dry ice and 37°C water bath, followed by clarification by centrifugation. Clarified lysates were then incubated with 5 mM dGTP, 100 μM compounds, or equivolume of DMSO (1%) at 37°C for 0.5-1 h, before being heated at increasing temperatures (CETSA) or 45°C (isothermal single-dose fingerprint CETSA) for 3 min. Following heating, samples were rested at RT for 3 min, clarified by centrifugation, and finally analysed by Western blot. Protein band intensities were normalised to that of the thermally stable loading control SOD-1, and were curve-fitted *via* a Boltzmann sigmoidal model (GraphPad Prism) to determine T_agg_.

##### Drug affinity responsive target stability

Drug affinity responsive target stability (DARTS) assay on THP-1 cell lysates was adapted from a previously reported method.^75^ THP-1 cells in logarithmic growth phase were collected, washed twice by PBS, and then lysed for 10 min at RT in M-PER™ mammalian protein extraction reagent (Cat.# 78501, Thermo Scientific) supplemented with 1X cOmplete, Mini EDTA-free protease inhibitor cocktail and Halt™ phosphatase inhibitor cocktail (Cat.# 78426, Thermo Scientific). Lysates were then clarified by centrifugation at 16000 x g for 10 min at 4°C. Protein concentrations of the clarified lysates were subsequently determined by Pierce™ Coomassie plus (Bradford) assay reagent (Thermo Scientific), and samples were diluted in 1x TN buffer (50 mM Tris-HCl pH 8.0, 50 mM NaCl) to a total protein concentration of 1 mg/mL. Thereafter, cell lysates were incubated with compounds or equivolume of DMSO (1%) at RT for 1 h, followed by digestion with desired concentration of pronase (Roche) solution, or 1XTN buffer only for non-digested samples, at RT for 30 min. Digestion was stopped by the addition of 4x Laemmli sample buffer (Bio-Rad) supplemented with 100 mM DTT and heating at 95°C for 5 min. Samples were then analysed by Western blot, where protein band intensities were normalised to that of SOD-1, and further compared to non-digested samples.

#### Western blot

Centrifugation-clarified cell lysates were mixed in β-mercaptoethanol-supplemented Laemmli buffer (Bio-Rad) and then heated at 99°C for 5-10 min. Samples were subject to sodium dodecyl sulfate-polyacrylamide gel electrophoresis (SDS-PAGE) using 4–15% Mini-PROTEAN TGX gels, and proteins were subsequently transferred to nitrocellulose membranes using a Trans-Blot Turbo machine (Bio-Rad). Following blocking with Odyssey Blocking Buffer (LI-COR), membranes were first probed with primary antibodies at RT for 1 h or 4°C overnight, and then probed with species-appropriate secondary antibodies at RT for 30 min. Between incubations, membranes were washed trice using TBST (Tris-buffered saline, 0.1% Tween 20). Protein bands were visualised using an Odyssey Fc Imaging System (Li-Cor Biosciences), and subsequently analysed using Image Studio Lite (Ver. 5.2, Li-Cor Biosciences). All uncropped western blot images are provided in the source data.

#### Proliferation inhibition assay

Proliferation inhibition assay was conducted as described previously.^52^ Briefly, compounds of indicated concentrations were spotted into 384-well plates using a D300e Digital Dispenser (Tecan). When applicable, DMSO levels were normalised across the plate at the maximum level of 1%. Cells were then seeded into the compound-containing plates using a Multidrop Combi Reagent Dispenser (Thermo Fisher Scientific), at 1000 cells/well in 50 μL medium (serum levels were kept at 5% to facilitate accurate dispensing). Cells were then incubated at 37°C and 5% CO_2_ in a humidified incubator for 72-96 h, before being incubated with resazurin reagent (final concentration - 0.01 mg/mL for another 4-6 h). Fluorescence signals were subsequently acquired using 530/590 nm (ex/em) filters on a Hidex Sense Microplate Reader and were used to determine relative cell viabilities by normalising to cell-only (100% viability) and medium-only (0% viability) wells. Relative cell viabilities were further curve-fitted using a nonlinear regression model (variable slope, four-parameter, GraphPad Prism), when applicable.

#### Chemical synthesis

All reagents and solvents were purchased from Sigma-Aldrich, Combi-Blocks, Thermo Fischer Scientific, or VWR and were used without purification. Unless otherwise stated, reactions were performed without care to exclude air or moisture. Analytical thin-layer chromatography was performed on silica gel 60 F-254 plates (E. Merck) and visualized under an UV lamp. Flash column chromatography was performed in a Biotage® SP4 MPLC system using Merck silica gel 60 Å (40–63 μm mesh). ^1^H and ^13^C NMR spectra were recorded on Bruker DRX-400 MHz and Bruker Avance 400 spectrometer. Chemical shifts are expressed in parts per million (ppm) and referenced to the residual solvent peak. For ^1^H and ^13^C measurements, the chemical shift is referred to an internal standard; the remaining protons or respectively the carbons of the corresponding deuterated solvent were used. Analytical LC–MS were performed on an Agilent MSD mass spectrometer connected to an Agilent 1100 system with: Method ST1090A3: Column ACE 3 C8 (50 × 3.0 mm); H_2_O (+ 0.1% TFA) and MeCN were used as mobile phases at a flow rate of 1 ml/min, with a gradient from 10% – 90% in 3 min; or Method B0597X3: Column Xterra MSC18 (50 × 3.0 mm); H_2_O (containing 10 mM NH_4_HCO_3_; pH = 10) and MeCN were used as mobile phases at a flow rate of 1 ml/min, with a gradient of 5% – 97% in 3 min. For LC–MS, detection was made by UV (254 or 214 nm) and MS (ESI+). Preparative LC was performed on a Gilson system using Waters C18 OBD 5 μm column (30 × 75 mm) with water buffer (a) 50 mM NH_4_HCO_3_ at pH 10 or b) 0.1% TFA) and acetonitrile as mobile phases using a flow rate of 45 ml/min. All final compounds were assessed to be >95% pure by LC–MS analysis.

For 6-bromo-N-(2-(pyridin-2-yl)ethyl)imidazo[1,2-a]pyrazin-8-amine, 250 mg (0.9 mmol) of 6,8-dibromoimidazo[1,2-a]pyrazine were dissolved in 5 mL Isopropanol and 1.1 eq. (119 μL, 0.99 mmol) 2-(pyridin-2-yl)ethanamine and 2 eq. (315 μL, 1.8 mmol) DIPEA were added. The reaction was stirred at 100°C overnight. The reaction mixture was evaporated and the crude was used for the synthesis of below compounds. Alternatively, the precursor can be purified by silica column chromatography (10 g silica per 200 mg crude, ethyl acetate/isopropanol, gradient 0 to 5 %). The crude was dry-loaded onto silica in ethyl acetate/methanol (1:1). Target compound elutes at 3 % isopropanol in ethyl acetate. C13H12BrN5, M = 318.17 g/mol, LC-MS: [M+H]^+^ 319; ^1^H-NMR (MeOD, 400 MHz): δ 8.34 (d, J = 4.67 Hz, 1H), 7.70 (s, 1H), 7.59 (dt, J_1_ = 7.80 Hz, J_2_ = 1.77 Hz, 1H), 7.57 (d, J = 1.01 Hz, 1H), 7.32 (d, J = 1.01 Hz, 1H), 7.21 (d, J = 7.80 Hz, 1H), 7.12 (td, J_1_ = 6.32 Hz, J_2_ = 0.90 Hz, 1H), 3.79 (t, J = 6.94 Hz, 2H), 3.04 (t, J = 6.94 Hz, 2H); ^13^C-NMR (MeOD, 100 MHz): 159.05, 148.31, 147.11, 137.19, 131.81, 131.85, 123.80, 122.48, 121.71, 115.39, 109.24, 40.21, 36.61; TLC (Ethyl acetate/isopropanol, 9:1): R_f_ = 0.37.

For 6-(2-chlorophenyl)-N-(2-(pyridin-2-yl)ethyl)imidazo[1,2-a]pyrazin-8-amine (TH6342), 30 mg (0.094 mmol) of 6-bromo-N-(2-(pyridin-2-yl)ethyl)imidazo[1,2-a]pyrazin-8-amine was dissolved in 1 mL dioxane. Subsequently, 1.5 eq. (22.1 mg, 0.14 mmol) of (2-chlorophenyl)boronic acid, 0.2 eq. (21.8 mg, 0.018 mmol) tetrakis(triphenylphosphin)-palladium(0) and 126 μL of a 1 mM Na_2_CO_3_ solution were added and the reaction was refluxed overnight. The reaction mixture was cooled to room temperature and filtered through Celite using MeOH. The solvents were evaporated and the crude product was purified over RP-HPLC to afford the desired product as a brown oil (3.3 mg, 9%). Alternative purification by silica column chromatography (Ethyl acetate/isopropanol, 10 g silica per 0.05 mmol substrate). The reaction crude was dry-loaded onto silica in acetone. Target compound elutes at 3 % isopropanol in ethyl acetate. C19H16ClN5, M = 349.104 g/mol; LCMS: [M+H]^+^ 350; ^1^H-NMR (400 MHz, CDCl_3_, δ = 7.27): 11.17 (brs, 2H), 9.04 (brs, 1H), 8.67 (dd, J = 5.7 Hz, 0.9 Hz, 1H), 8.13 (td, J = 7.8 Hz, 7.8 Hz, 1.6 Hz, 1H), 7.88 (s, 1H), 7.74-7.67 (m, 4H), 7.57-7.53 (m, 1H), 7.51-7.49 (m, 1H), 7.45-7.37 (m, 2H), 4.19 (brs, 2H), 3.50-3.47 (m, 2H) ppm.; ^13^C-NMR-DEPT (101 MHz, CDCl_3_, δ = 77.2): 143.6, 142.6, 131.9, 130.3, 127.3, 126.8, 124.0, 109.7, 40.6, 33.6 ppm.

For 6-(2-aminophenyl)-N-(2-(pyridin-2-yl)ethyl)imidazo[1,2-a]pyrazin-8-amine (TH7126), 30 mg (0.094 mmol) of 6-bromo-N-(2-(pyridin-2-yl)ethyl)imidazo[1,2-a]pyrazin-8-amine was dissolved in 1 mL dioxane. Subsequently, 1.5 eq. (31.0 mg, 0.14 mmol) of 2-(4,4,5,5-tetramethyl-1,3,2-dioxaborolan-2-yl)aniline, 0.2 eq. (21.8 mg, 0.018 mmol) tetrakis-(triphenylphosphin)palladium(0) and 126 μL of a 1mM Na_2_CO_3_ solution were added and the reaction was refluxed overnight. The reaction mixture was cooled to room temperature and filtered through Celite using MeOH. The solvents were evaporated and the crude product was purified over RP-HPLC to afford the desired product as an orange solid (22.2 mg, 71%). C19H18N6, M = 330.1593 g/mol; LCMS: [M+H]^+^ 331; ^1^H-NMR (400 MHz, CDCl_3_, δ = 7.27): 8.59-8.57 (m, 1H), 7.62 (s, 1H), 7.59 (td, J = 7.7 Hz, 7.7 Hz, 1.8 Hz, 1H), 7.51 (d, J = 0.6 Hz, 2H), 7.33 (dd, J = 7.9 Hz, 1.1 Hz, 1H), 7.20-7.12 (m, 3H), 6.80-6.76 (m, 2H), 6.66 (t, J = 5.5 Hz, 1H), 4.58 (brs, 2H), 4.05 (q, J = 6.6 Hz, 2H), 3.19 (t, J = 6.6 Hz, 2H) ppm. ; ^13^C-NMR (101 MHz, CDCl_3_, δ = 77.2): 159.1, 149.4, 147.3, 146.1, 140.1, 136.6, 132.1, 132.0, 129.4, 128.8, 123.4, 122.0, 121.6, 118.1, 116.9, 115.0, 108.0, 40.2, 37.4 ppm.

For 6-(2-methoxyphenyl)-N-(2-(pyridin-2-yl)ethyl)imidazo[1,2-a]pyrazin-8-amine (TH7127), 30 mg (0.094 mmol) of 6-bromo-N-(2-(pyridin-2-yl)ethyl)imidazo[1,2-a]pyrazin-8-amine was dissolved in 1 mL dioxane. Subsequently, 1.5 eq. (21.5 mg, 0.14 mmol) of (2-methoxyphenyl)boronic acid, 0.2 eq. (21.8 mg, 0.018 mmol) tetrakis-(triphenylphosphin)palladium(0) and 126 μL of a 1mM Na_2_CO_3_ solution were added and the reaction was refluxed overnight. The reaction mixture was cooled to room temperature and filtered through Celite using MeOH. The solvents were evaporated and the crude product was purified over RP-HPLC to afford the desired product as a green solid (11.7 mg, 34%). Alternative purification by silica column chromatography (Ethyl acetate/isopropanol, 10 g silica per 0.1 mmol substrate). Target compound elutes at 10 % isopropanol in ethyl acetate. C20H19N5O, M = 345.1590 g/mol; LCMS: [M+H]^+^ 346; ^1^H-NMR (400 MHz, CDCl_3_, δ = 7.27): 13.58 (brs, 2H), 8.98 (brs, 1H), 8.63 (d, J = 5.4 Hz), 8.42 (brs, 1H), 8.2 (td, J = 7.9 Hz, 7.9 Hz, 1.3 Hz, 1H), 8.05 (brs, 1H), 7.81 (d, J = 7.9 Hz), 7.73-7.71 (m, 2H), 7.60 (t, J = 6.5 Hz, 6.5 Hz, 1H), 7.41-7.37 (m, 1H), 7.09 (m, 1H), 7.00 (m, 1H), 4.23 (brs, 2H), 3.15 (s, 3H), 3.54-3.51 (m, 2H) ppm; ^13^C-NMR-DEPT (101 MHz, CDCl_3_, δ = 77.2): 144.8, 141.9, 130.8, 130.7, 127.4, 124.4, 121.1, 111.3, 110.3, 55.6, 40.3, 33.2 ppm.

For N-(2-(pyridin-2-yl)ethyl)-6-(thiophen-2-yl)imidazo[1,2-a]pyrazin-8-amine (TH7528), 27.5 mg (0.086 mmol) of 6-bromo-N-(2-(pyridin-2-yl)ethyl)imidazo[1,2-a]pyrazin-8-amine was dissolved in 1 mL dioxane. Subsequently, 1.5 eq. (16.6 mg, 0.13 mmol) of thiophen-2-yl boronic acid, 0.2 eq. (20.0 mg, 0.017 mmol) tetrakis(triphenylphosphin)palladium(0) and 115 μL of a 1mM Na_2_CO_3_ solution were added and the reaction was refluxed overnight. The reaction mixture was cooled to room temperature and filtered through Celite using MeOH. The solvents were evaporated and the crude product was purified over RP-HPLC to afford the desired product as a yellow oil (11.7 mg, 34%). C17H15N5S g/mol, M = 321.1048; LCMS: 322; ^1^H-NMR (400 MHz, CDCl_3_, δ = 7.27): 8.68-8.66 (m, 1H), 7.83 (s, 1H), 7.81-7.79 (m, 1H), 7.54-7.53 (m, 2H), 7.48-7.45 (m, 2H), 7.35 (dd, J = 5.1 Hz, 1.1 Hz, 1H), 7.33-7.29 (m, 1H), 7.11-7.09 (m, 1H), 4.17-4.13 (m, 2H), 3.39 (t, J = 6.6 Hz, 2H) ppm; ^13^C-NMR-DEPT (101 MHz, CDCl_3_, δ = 77.2): 146.9, 139.3, 127.9, 126.3, 124.9, 123.1, 122.4, 115.2, 40.5, 35.8 ppm.

### Quantification and statistical analysis

Statistical analysis was conducted using GraphPad Prism 9 software. Specific statistical test details, including test method, n values (number of independent experiments), number of technical repeats per independent experiment, and dispersion and precision measures, are indicated in the corresponding figure legends. Statistical significance is defined as p < 0.05, unless otherwise stated. Asterisk in figures signifies statistical significance (∗ for p ≤ 0.05, ∗∗ for p ≤ 0.01, ∗∗∗ for p ≤ 0.001, ∗∗∗∗ for p ≤ 0.0001).

## Supplemental information

Document S1. Figures S1–S14 and Tables S1 and S3

Table S2. Data of primary screening campaign, and the subsequent three-point (2.5, 10 and 40 μM) dose-response validation, related to Figure 1
