# Supplementary material for: Identification and evaluation of small-molecule inhibitors against the dNTPase SAMHD1 via a comprehensive screening funnel
Source: iScience. 2024 Jan 13;27(2):108907. doi: 10.1016/j.isci.2024.108907 (PMC10839966; doi:10.1016/j.isci.2024.108907)
Supplement: Document S1. Figures S1–S14 and Tables S1 and S3 [file mmc1.pdf]

iScience, Volume *27*

Supplemental information

Identification and evaluation of small-molecule inhibitors against the dNTPase SAMHD1 via a comprehensive screening funnel

Si Min Zhang, Cynthia B.J. Paulin, Huazhang Shu, Miriam Yagüe-Capilla, Maurice Michel, Petra Marttila, Florian Ortis, Henri Colyn Bwanika, Christopher Dirks, Rajagopal Papagudi Venkatram, Elisée Wiita, Ann-Sofie Jemth, Ingrid Almlöf, Olga Loseva, Femke M. Hormann, Tobias Koolmeister, Erika Linde, Sun Lee, Sabin Llona-Minguez, Martin Haraldsson, Hanna Axelsson, Kia Strömberg, Evert J. Homan, Martin Scobie, Thomas Lundbäck, Thomas Helleday, and Sean G. Rudd

Figure S1. Supplemental information of the high-throughput screening campaign for SAMHD1 inhibitors, related to Figure 1.

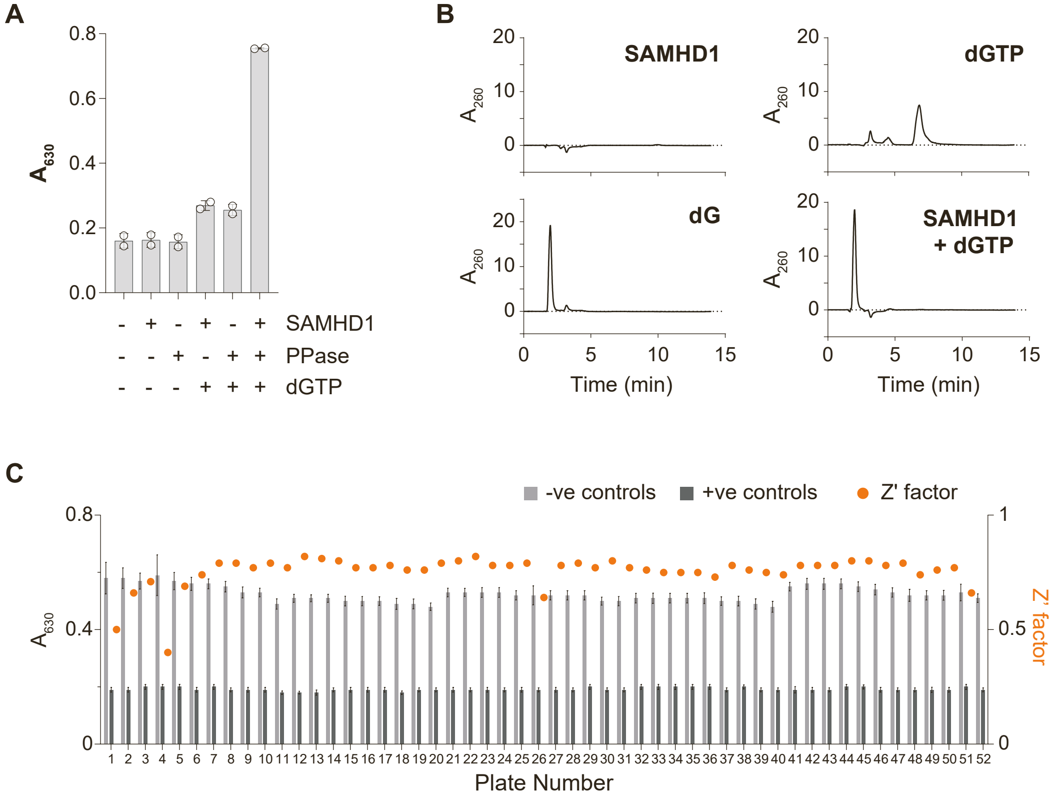

A. SAMHD1 hydrolytic activity in MG enzyme-coupled assay required the presence of SAMHD1, PPase, and dGTP. Mean absorbance at 630 nm ± SD of n = 2 independent experiments each performed in duplicate are shown. **B.** SAMHD1-mediated hydrolysis of dGTP into dG, with reaction products confirmed using high performance liquid chromatography (HPLC). HPLC profiles of a representative experiment are shown. **C.** Plotted values of the positive control, negative control, and z' factor for each plate of the high-throughput screening campaign. Absorbance at 630 nm (A630nm) of positive (SAMHD1-free, represents 100% inhibition) and negative (SAMHD1 only without screening compounds, represents 0% inhibition) controls are plotted on the left axis, and z' factor values are plotted on the right axis. Mean A630nm ± SD of 26 positive or negative control wells included in each plate are shown.

Figure S2. Validation of the high-throughput screening hits, related to Figure 1.

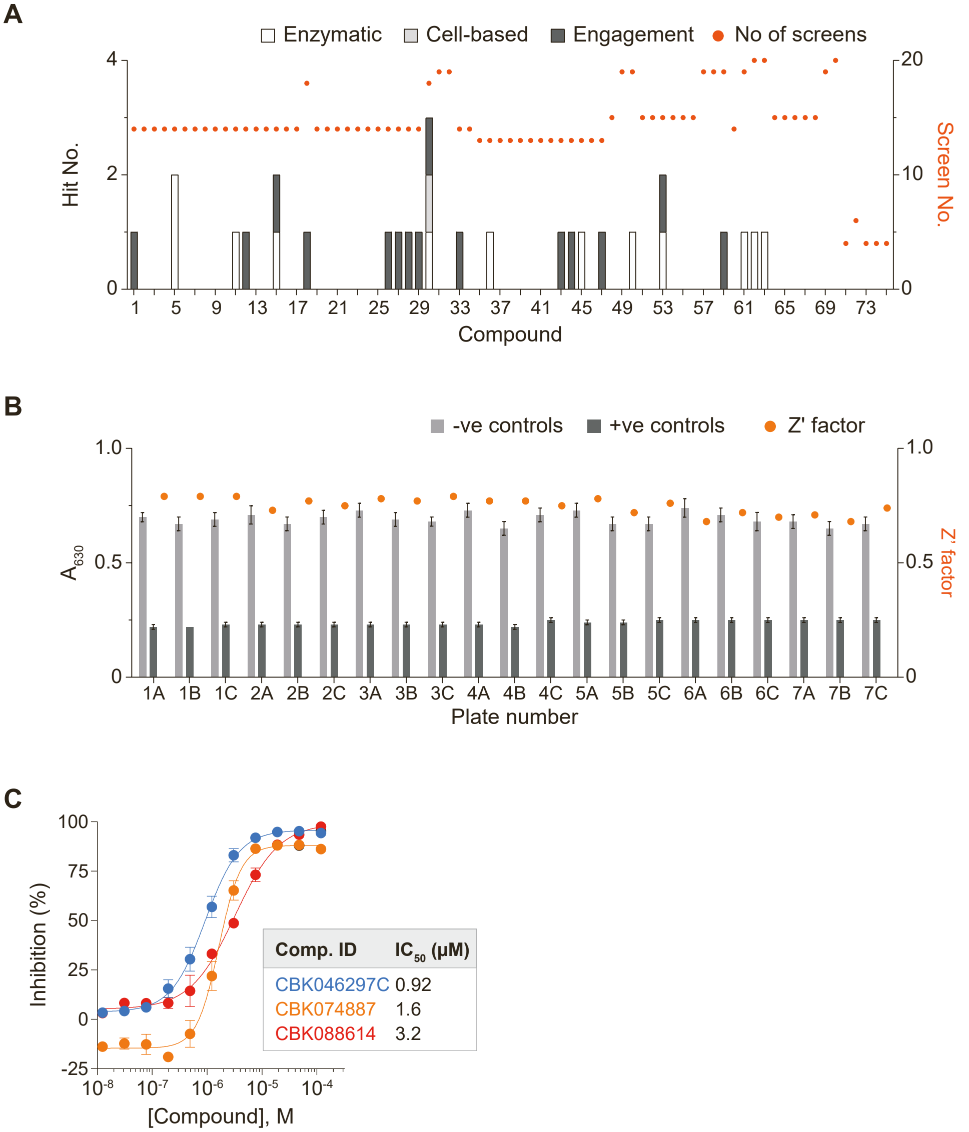

A. Exclusion of hit compounds displayed promiscuity in previous in-house screening campaigns. For each putative SAMHD1 inhibitor hit, number of times this compound was screened against other targets is plotted on the right axis, and number times this compound was identified as a hit is potted on the left axis. Screening campaigns were summarised based on assay types, i.e., enzymatic, cell-based or engagement assay. **B.** Plotted values of the positive control, negative control, and z' factor for each plate of the 11-point dose-response hit validation. A630nm of positive (SAMHD1-free, represents 100% inhibition) and negative (SAMHD1 only without screening compounds, represents 0% inhibition) controls are plotted on the left axis, and z' factor values are plotted on the right axis. Mean A630nm ± SD of 16 positive or negative control wells included in each plate are shown. **C.** Representative dose-response curve validation of hit compounds. Recombinant SAMHD1 was incubated with increasing concentrations of hit compounds, and enzymatic activities were determined using enzyme-coupled MG assay. Mean inhibition % ± SD of a representative experiment performed in triplicate are shown. The half maximal inhibitory concentrations (IC50) were determined by curve-fitting inhibition % using a non-linear regression model (variable slope, four parameters, GraphPad Prisim).

Figure S3. Structure activity relationship (SAR) study of TH6342 and analogues, related to Figure 2.

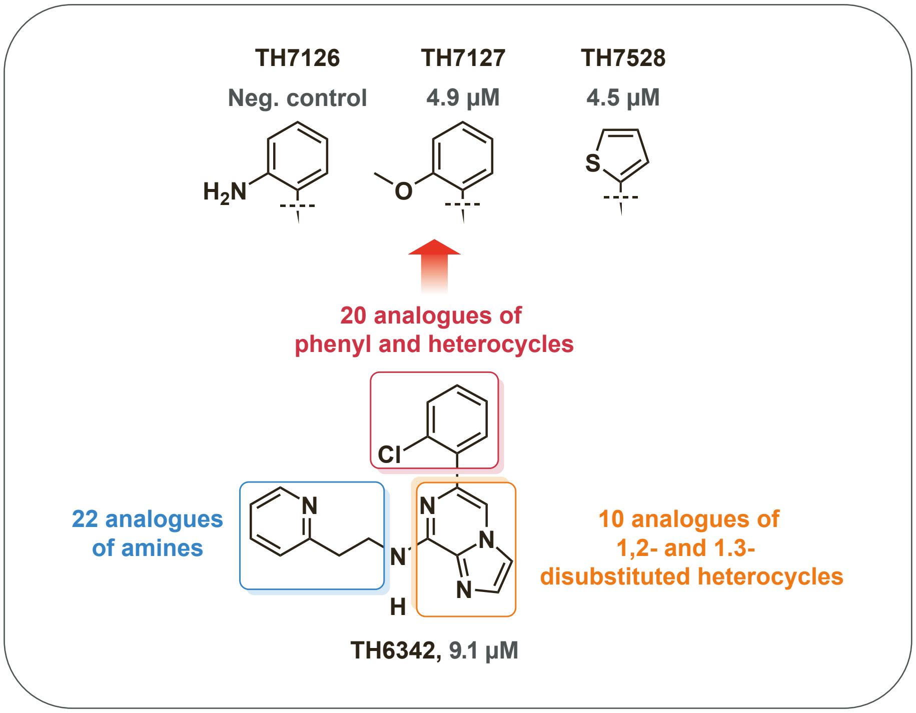

Figure S4. Supplemental information of DSF experiments on recombinant SAMHD1 protein incubated with nucleotide(s) or SAMHD1 inhibitors, related to Figure 3.

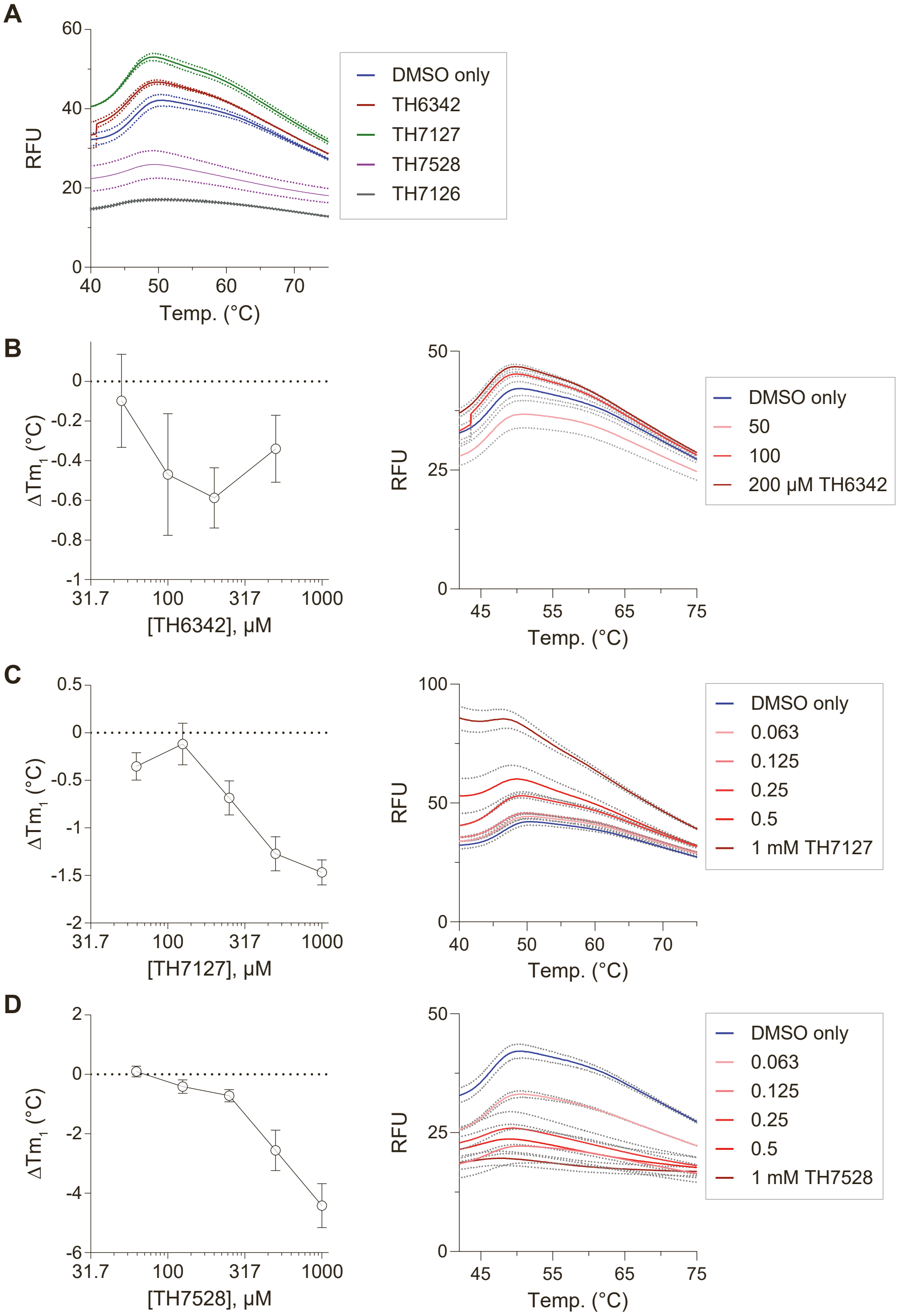

A. Melting curves of recombinant SAMHD1 protein in the presence or absence of putative SAMHD1 inhibitors. Recombinant SAMHD1 protein was incubated with 200-250 μM TH6342, TH7127 and TH7528 or equal volume of DMSO, before its thermal stability being examined by DSF. Mean fluorescence signals (solid line) ± SEM (dashed line) of a representative experiment performed in triplicate are shown. **B-D.** TH6342 (B), TH7127 (C) and TH7528 (D) reduced the Tm1 of recombinant SAMHD1 in a dose-dependent manner. Left panels, mean ΔTm1 ± SEM of n ≥ 2 independent experiments performed in triplicates or quadruplicates are shown. Right panels, melting curves of recombinant SAMHD1 protein in the presence of TH6342, TH7127, or TH7528. Mean fluorescence signals (solid line)± SEM (dashed line) of a representative experiment performed in triplicate are shown.

Figure S5. Supplemental information of DSF experiments on recombinant SAMHD1 protein co-incubated with nucleotide(s) and TH6342/7127, related to Figure 3.

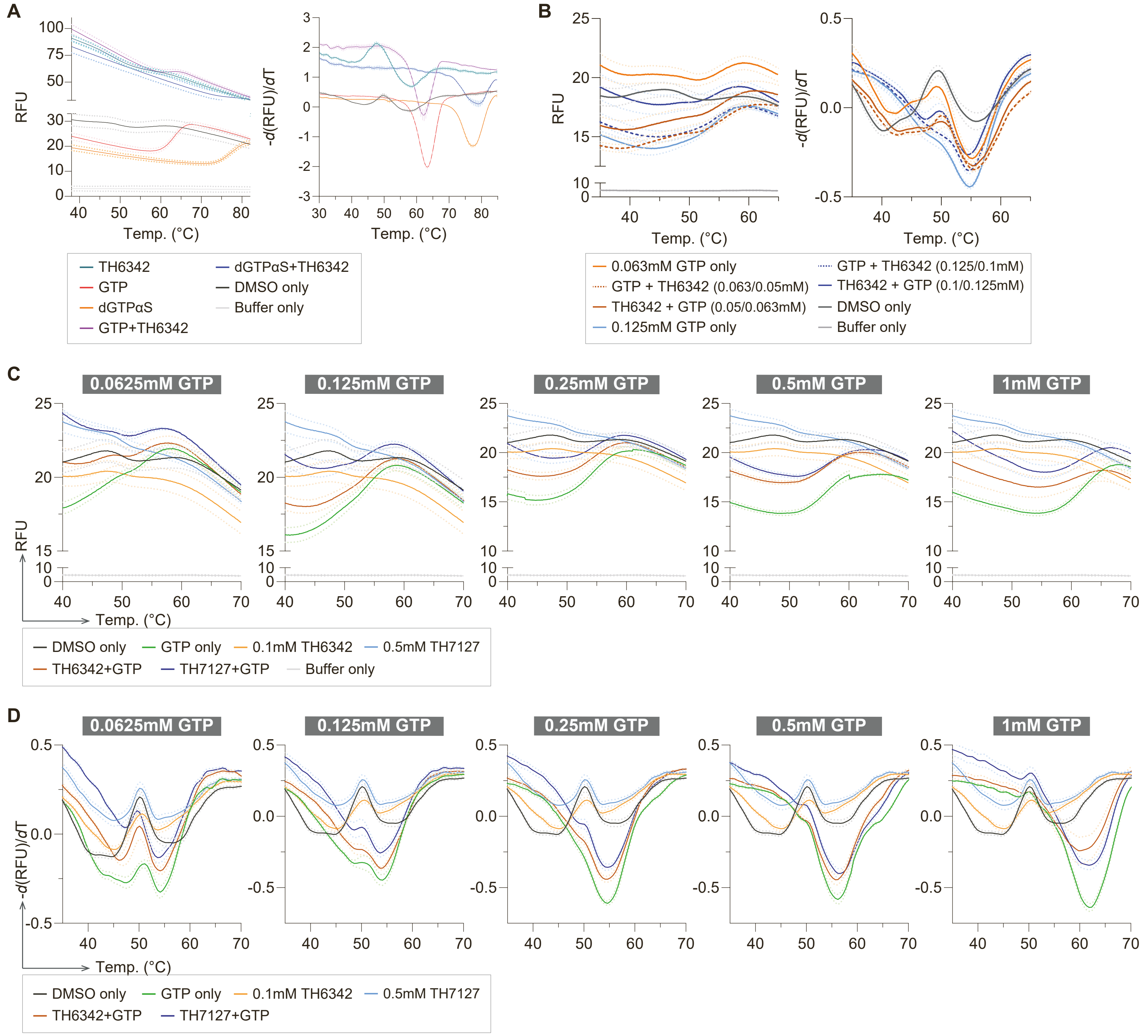

A. TH6342 at 0.5 mM decreased the Tm of recombinant SAMHD1 protein in the presence of GTP. Melting profile of recombinant SAMHD1 treated with activating nucleotides (1 mM GTP or 5 mM dGTPαS), alone or followed by 0.5 mM TH6342. Left panel, melting curves of recombinant SAMHD1 in different treatment groups. Mean fluorescence signals (solid line) ± SEM (dashed line) of a representative experiment performed in quadruplicate are shown. Right panel, negative derivative (-dRFU/dT) of the SAMHD1 melting curves shown in the left panel are shown. Mean negative derivative values (solid lines) ± SEM (dashed lines) of a representative experiment performed in quadruplicates are shown. **B.** Melting profile of recombinant SAMHD1 co-treated with GTP and TH6342, at alternating orders. Left panel, mean fluorescence signals (solid line) ± SEM (dashed line) of n = 2 independent experiments performed in quadruplicate are shown; right panel, mean negative derivative (-dRFU/dT) (solid line) ± SEM (dashed line) of the melting curves are shown. **C-D.** Melting profile of recombinant SAMHD1 treated with 0.1mM TH6342 or 0.5 mM TH7127, followed by increasing concentrations of GTP. In C, mean fluorescence signals (solid line) ± SEM (dashed line) of n = 1-2 independent experiments performed in quadruplicate are shown; in D, mean negative derivative (-dRFU/dT) (solid line) ± SEM (dashed line) of the melting curves are shown.

Figure S6. Supplemental information of DSF experiments on recombinant SAMHD1 protein co-incubated with nucleotide(s) and TH7126, related to Figure 3.

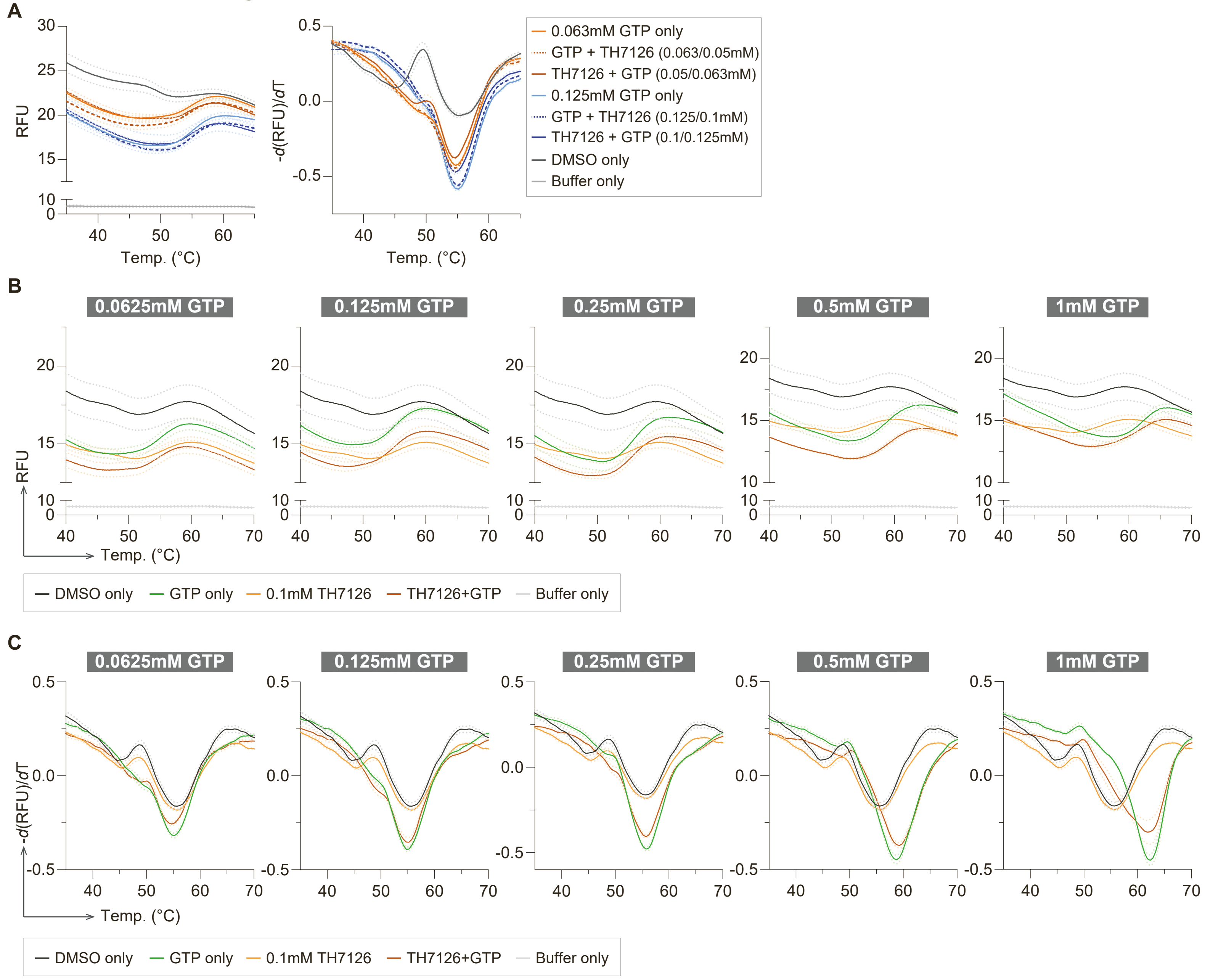

A. Melting profile of recombinant SAMHD1 co-incubated with GTP and TH7126, at alternating orders. Left panels, mean fluorescence signals (solid line) ± SEM (dashed line) of n = 2 independent experiments performed in quadruplicate are shown; right panel, mean negative derivative (-dRFU/dT) (solid line) ± SEM (dashed line) of the melting curves are shown. **B-C.** Melting profile of recombinant SAMHD1 incubated with 0.1mM TH7126, followed by increasing concentrations of GTP. In B, mean fluorescence signals (solid line) ± SEM (dashed line) of n = 2 independent experiments performed in quadruplicate are shown; in C, mean negative derivative (-dRFU/dT)(solid line) ± SEM (dashed line) of the melting curves are shown.

Figure S7. Supplemental information of recombinant SAMHD1 protein multimerization, assessed via DLS and in vitro chemical crosslinking, related to Figure 3.

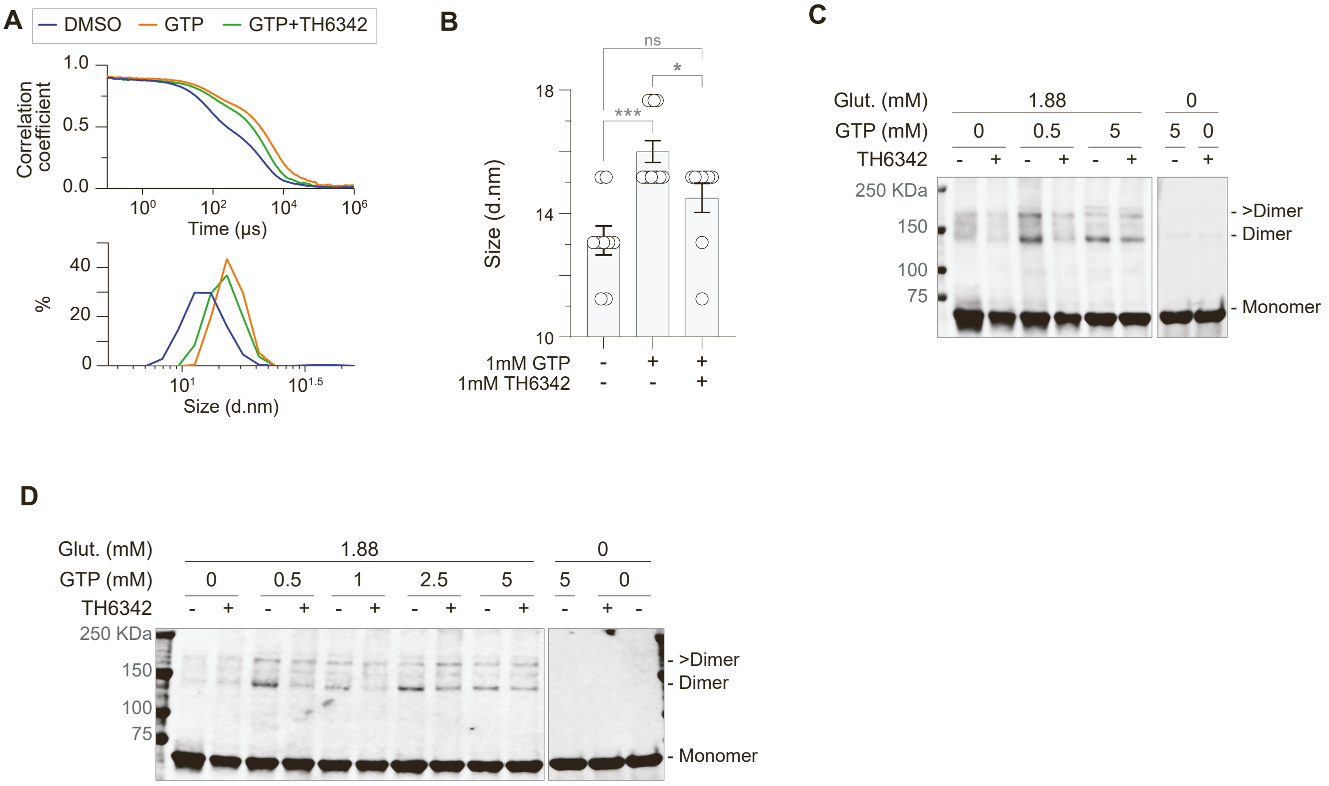

A-B. DLS measurements revealed TH6342 (1 mM) deterred GTP (1 mM)-induced SAMHD1 dimerization. In A, correlogram (top) and weight-average distribution (by volume) (bottom) of recombinant SAMHD1 of a representative experiment are shown; in B, estimated sizes (diameter, nm) of SAMHD1 under different conditions, where mean sizes of n = 3 independent experiments performed in duplicates to quadruplicates are shown, together with the individual repeat values. Ordinary one-way ANOVA (Turkey's multiple comparisons test) were performed between treatment groups, asterisk signifies statistical significance (* for $p \leq 0.05$, ** for $p \leq 0.01$, *** for $p \leq 0.001$, **** for $p \leq 0.0001$). Specifically, size (DMSO control) Vs. size (GTP only), $p = 0.0001$, $q = 6.929$, DF = 27; size (DMSO control) Vs. size (TH6342/GTP), $p = 0.09$, $q = 3.106$, DF = 27; size (GTP only) Vs. size (TH6342/GTP), $p = 0.043$, $q = 3.609$, DF = 27. **C-D.** In vitro chemical crosslinking revealed that TH6342 deterred GTP-induced SAMHD1 multimerization. Recombinant SAMHD1 was incubated with glutaraldehyde and/or indicated concentrations of GTP and/or TH6342, before formed protein multimers were separated by SDS-PAGE, followed by Western Blot. DMSO levels are controlled across treatment groups. Glutaraldehye concentration was set at 1.875mM to minimize unspecific oligomer formation in DMSO-only control groups, but allow stabilization of formed oligomers in GTP-treated groups. Protein species with sizes corresponding to SAMHD1 monomer, dimer and dimer-to-tetramer intermediates were indicated. Two representative experiments using different GTP concentration ranges are shown.

Figure S8. Supplemental information of kinetic studies using the enzyme-coupled MG activity assay, related to Figure 4.

A

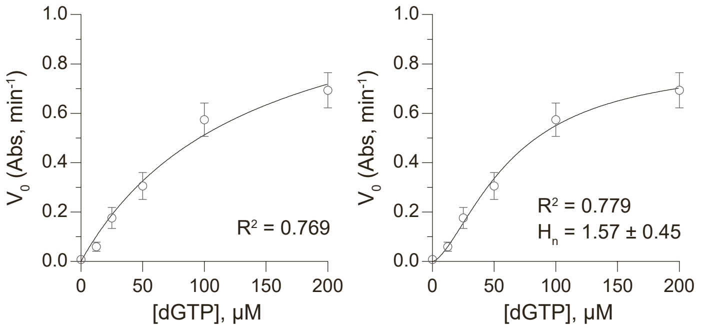

B

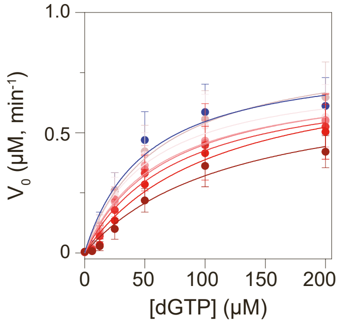

[TH6342]	Michaelis-Menten	Sigmoidal
20	0.8195	0.9993
10	0.7798	0.9987
3.3	0.7558	0.9992
1	0.8157	0.9953
0.3	0.7926	0.9982
0.1	0.7419	0.9995
0.03	0.7708	0.9986
0 μM	0.7729	0.9991

C

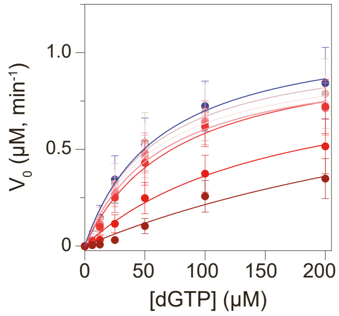

[TH7127]	Michaelis-Menten	Sigmoidal
20	0.7269	0.9995
10	0.7490	0.9969
3.3	0.8178	0.9992
1	0.8196	0.9996
0.3	0.8289	0.9985
0.1	0.7682	1.0000
0.03	0.7516	0.9996
0 μM	0.7787	0.9981

D

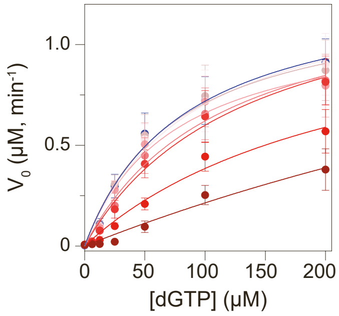

[TH7528]	Michaelis-Menten	Sigmoidal
20	0.8022	0.9990
10	0.8752	0.9973
3.3	0.8825	0.9997
1	0.8580	0.9990
0.3	0.8356	0.9992
0.1	0.8614	0.9976
0.03	0.8441	0.9984
0 μM	0.8886	0.9956

E

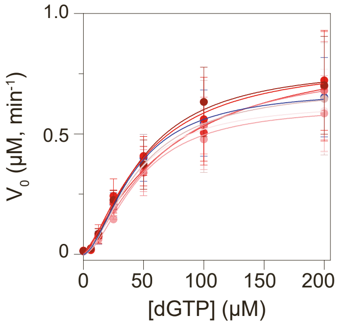

[TH7126]	Michaelis-Menten	Sigmoidal
20	0.8947	0.9939
10	0.8785	0.9943
3.3	0.8768	0.9942
1	0.8948	0.9988
0.3	0.8863	0.9965
0.1	0.8784	0.9989
0.03	0.8317	0.9987
0 μM	0.8936	0.9984

A. In the absence of putative inhibitors, SAMHD1 displayed minimal cooperativity in the enzyme-coupled MG activity assay with 25 μM dGTP as the self-activating substrate. Rate of reaction was fitted with either the Michaelis-Menten model (left panel) or an allosteric sigmoidal model (right panel), using GraphPad Prism. Goodness of fit parameter R2, as well as Hill coefficient (Hn) are shown. **B-E.** In the presence of SAMHD1 inhibitors, SAMHD1 displayed increasing levels of cooperativity in the enzyme-coupled MG activity assay with 25 μM dGTP as the self-activating substrate. Left panels, rate of reaction from Fig. 4A-D were re-fitted with the Michaelis-Menten model using GraphPad Prism. Right panels, goodness of fit parameter R2 from fitting with the Michaelis-Menten model were compared with those from fitting with an allosteric sigmoidal model as displayed in Fig. 4A-D.

Figure S9. Hill coefficient (Hn) values of reaction from Fig. 4A-D, related to Figure 4.

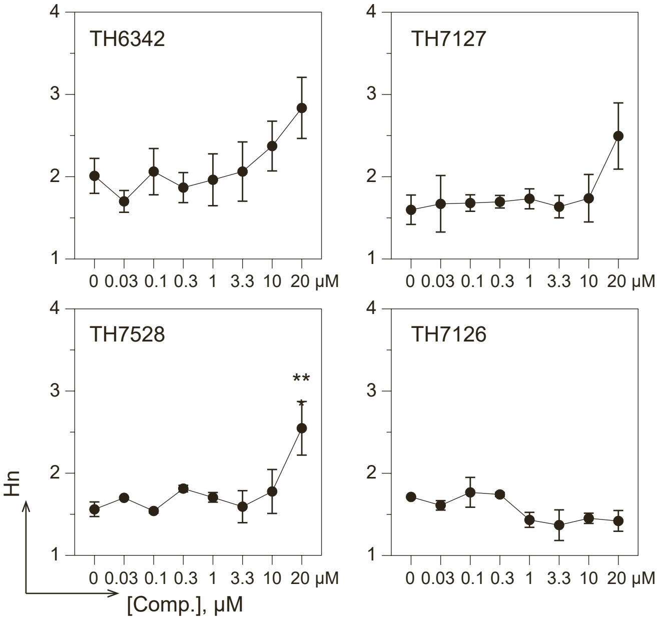

Hill coefficient was determined with an allosteric sigmoidal model (GraphPad Prism). Mean ± SEM of n = 3 independent experiments are shown. Ordinary one-way ANOVA (Dunnett's multiple comparisons test) were performed between nH in compound- versus DMSO-treated groups, asterisk signifies statistical significance (* for $p \le 0.05$, ** for $p \le 0.01$, *** for $p \le 0.001$, **** for $p \le 0.0001$). For TH6342, nH (0 μM) Vs. nH (0.03μM), p = 0.9466, q = 0.7784, DF = 16; nH (0 μM) Vs. nH (0.1μM), p = 0.9998, q = 0.1309, DF = 16; nH (0 μM) Vs. nH (0.33 μM), p = 0.9994, q = 0.3565, DF = 16; nH (0 μM) Vs. nH (1.1 μM), p = 0.9999, q = 0.1174, DF = 16; nH (0 μM) Vs. nH (3.33 μM), p = 0.9998, q = 0.1334, DF = 16; nH (0 μM) Vs. nH (10 μM), p = 0.8945, q = 0.9135, DF = 16; nH (0 μM) Vs. nH (20 μM), p = 0.2298, q = 2.079, DF = 16. For TH7127, nH (0 μM) Vs. nH (0.03μM), p = 0.9997, q = 0.2036, DF = 16; nH (0 μM) Vs. nH (0.1μM), p = 0.9997, q = 0.2273, DF = 16; nH (0 μM) Vs. nH (0.33 μM), p = 0.9996, q = 0.3041, DF = 16; nH (0 μM) Vs. nH (1.1 μM), p = 0.9977, q = 0.4156, DF = 16; nH (0 μM) Vs. nH (3.33 μM), p = 0.9999, q = 0.1177, DF = 16; nH (0 μM) Vs. nH (10 μM), p = 0.9975, q = 0.4375, DF = 16; nH (0 μM) Vs. nH (20 μM), p = 0.0703, q = 2.801, DF = 16. For TH7528, nH (0 μM) Vs. nH (0.03μM), p = 0.9883, q = 0.5779, DF = 16; nH (0 μM) Vs. nH (0.1μM), p = 0.9999, q = 0.0847, DF = 16; nH (0 μM) Vs. nH (0.33 μM), p = 0.8233, q = 1.050, DF = 16; nH (0 μM) Vs. nH (1.1 μM), p = 0.9848, q = 0.6043, DF = 16; nH (0 μM) Vs. nH (3.33 μM), p = 0.9998, q = 0.1320, DF = 16; nH (0 μM) Vs. nH (10 μM), p = 0.9006, q = 0.9001, DF = 16; nH (0 μM) Vs. nH (20 μM), p = 0.0047, q = 4.108, DF = 16. For TH7126, nH (0 μM) Vs. nH (0.03μM), p = 0.9769, q = 0.6542, DF = 16; nH (0 μM) Vs. nH (0.1μM), p = 0.9995, q = 0.3460, DF = 16; nH (0 μM) Vs. nH (0.33 μM), p = 0.9997, q = 0.1845, DF = 16; nH (0 μM) Vs. nH (1.1 μM), p = 0.3710, q = 1.768, DF = 16; nH (0 μM) Vs. nH (3.33 μM), p = 0.1982, q = 2.168, DF = 16; nH (0 μM) Vs. nH (10 μM), p = 0.4431, q = 1.640, DF = 16; nH (0 μM) Vs. nH (20 μM), p = 0.3332, q = 1.841, DF = 16.

Figure S10. Fold changes (%) of Vmax and $K_{0.5}$ of reactions from Fig. 4A-D, related to Figure 4.

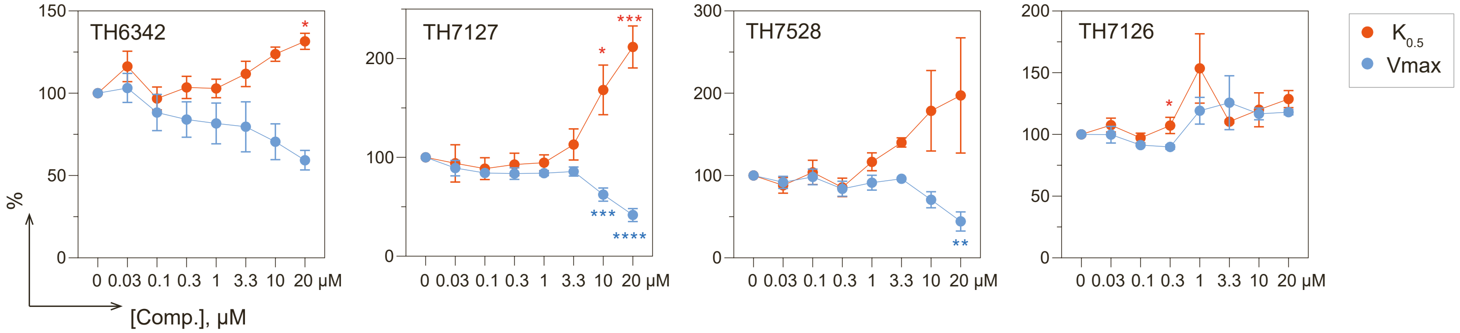

SAMHD1 inhibitors reduced the reaction Vmax, but increased $K_{0.5}$, as determined by fitting rate of reaction from Fig. 4A-D with an allosteric sigmoidal model (GraphPad Prism). Mean ± SEM of n = 3 independent experiments are shown. Fold changes (%) in Vmax and $K_{0.5}$ are shown. Ordinary one-way ANOVA (Dunnett's multiple comparisons test) were performed between compound- versus DMSO-treated groups, asterisk signifies statistical significance (* for p ≤ 0.05, ** for p ≤ 0.01, *** for p ≤ 0.001, **** for p ≤ 0.0001). For % $K_{0.5}$ comparisons – for TH6342, % $K_{0.5}$ (0 µM) Vs. % $K_{0.5}$ (0.03µM), p = 0.3344, q = 1.838, DF = 16; % $K_{0.5}$ (0 µM) Vs. % $K_{0.5}$ (0.1µM), p = 0.9994, q = 0.3559, DF = 16; % $K_{0.5}$ (0 µM) Vs. % $K_{0.5}$ (0.33 µM), p = 0.9978, q = 0.3977, DF = 16; % $K_{0.5}$ (0 µM) Vs. % $K_{0.5}$ (1.1 µM), p = 0.9995, q = 0.3367, DF = 16; % $K_{0.5}$ (0 µM) Vs. % $K_{0.5}$ (3.33 µM), p = 0.6423, q = 1.331, DF = 16; % $K_{0.5}$ (0 µM) Vs. % $K_{0.5}$ (10 µM), p = 0.0789, q = 2.684, DF = 16; % $K_{0.5}$ (0 µM) Vs. % $K_{0.5}$ (20 µM), p = 0.0140, q = 3.565, DF = 16. For TH7127, % $K_{0.5}$ (0 µM) Vs. % $K_{0.5}$ (0.03µM), p = 0.9996, q = 0.2699, DF = 16; % $K_{0.5}$ (0 µM) Vs. % $K_{0.5}$ (0.1µM), p = 0.9940, q = 0.5071, DF = 16; % $K_{0.5}$ (0 µM) Vs. % $K_{0.5}$ (0.33 µM), p = 0.9995, q = 0.3205, DF = 16; % $K_{0.5}$ (0 µM) Vs. % $K_{0.5}$ (1.1 µM), p = 0.9997, q = 0.2402, DF = 16; % $K_{0.5}$ (0 µM) Vs. % $K_{0.5}$ (3.33 µM), p = 0.9877, q = 0.5832, DF = 16; % $K_{0.5}$ (0 µM) Vs. % $K_{0.5}$ (10 µM), p = 0.0387, q = 3.056, DF = 16; % $K_{0.5}$ (0 µM) Vs. % $K_{0.5}$ (20 µM), p = 0.0008, q = 4.999, DF = 16. For TH7528, % $K_{0.5}$ (0 µM) Vs. % $K_{0.5}$ (0.03µM), p = 0.9996, q = 0.2688, DF = 16; % $K_{0.5}$ (0 µM) Vs. % $K_{0.5}$ (0.1µM), p = 0.9999, q = 0.08883, DF = 16; % $K_{0.5}$ (0 µM) Vs. % $K_{0.5}$ (0.33 µM), p = 0.9995, q = 0.3247, DF = 16; % $K_{0.5}$ (0 µM) Vs. % $K_{0.5}$ (1.1 µM), p = 0.9994, q = 0.3759, DF = 16; % $K_{0.5}$ (0 µM) Vs. % $K_{0.5}$ (3.33 µM), p = 0.8976, q = 0.9067, DF = 16; % $K_{0.5}$ (0 µM) Vs. % $K_{0.5}$ (10 µM), p = 0.3673, q = 1.774, DF = 16; % $K_{0.5}$ (0 µM) Vs. % $K_{0.5}$ (20 µM), p = 0.1890, q = 2.196, DF = 16. For TH7126, % $K_{0.5}$ (0 µM) Vs. % $K_{0.5}$ (0.03µM), p = 0.9974, q = 0.4414, DF = 15; % $K_{0.5}$ (0 µM) Vs. % $K_{0.5}$ (0.1µM), p = 0.9998, q = 0.1627, DF = 15; % $K_{0.5}$ (0 µM) Vs. % $K_{0.5}$ (0.33 µM), p = 0.9976, q = 0.4246, DF = 15; % $K_{0.5}$ (0 µM) Vs. % $K_{0.5}$ (1.1 µM), p = 0.0374, q = 3.106, DF = 15; % $K_{0.5}$ (0 µM) Vs. % $K_{0.5}$ (3.33 µM), p = 0.9923, q = 0.5398, DF = 15; % $K_{0.5}$ (0 µM) Vs. % $K_{0.5}$ (10 µM), p = 0.7607, q = 1.161, DF = 15; % $K_{0.5}$ (0 µM) Vs. % $K_{0.5}$ (20 µM), p = 0.4353, q = 1.666, DF = 15. For % Vmax comparisons – for TH6342, % Vmax (0 µM) Vs. % Vmax (0.03µM), p = 0.9997, q = 0.220, DF = 16; % Vmax (0 µM) Vs. % Vmax (0.1µM), p = 0.9400, q = 0.7989, DF = 16; % Vmax (0 µM) Vs. % Vmax (0.33 µM), p = 0.7988, q = 1.092, DF = 16; % Vmax (0 µM) Vs. % Vmax (1.1 µM), p = 0.6934, q = 1.255, DF = 16; % Vmax (0 µM) Vs. % Vmax (3.33 µM), p = 0.5991, q = 1.396, DF = 16; % Vmax (0 µM) Vs. % Vmax (10 µM), p = 0.2530, q = 2.019, DF = 16; % Vmax (0 µM) Vs. % Vmax (20 µM), p = 0.0649, q = 2.788, DF = 16. For TH7127, % Vmax (0 µM) Vs. % Vmax (0.03µM), p = 0.5480, q = 1.473, DF = 16; % Vmax (0 µM) Vs. % Vmax (0.1µM), p = 0.1998, q = 2.163, DF = 16; % Vmax (0 µM) Vs. % Vmax (0.33 µM), p = 0.1756, q = 2.240, DF = 16; % Vmax (0 µM) Vs. % Vmax (1.1 µM), p = 0.1945, q = 2.179, DF = 16; % Vmax (0 µM) Vs. % Vmax (3.33 µM), p = 0.2793, q = 1.957, DF = 16; % Vmax (0 µM) Vs. % Vmax (10 µM), p = 0.0006, q = 5.135, DF = 16; % Vmax (0 µM) Vs. % Vmax (20 µM), p < 0.0001, q = 7.969, DF = 16. For TH7528, % Vmax (0 µM) Vs. % Vmax (0.03µM), p = 0.9666, q = 0.7048, DF = 16; % Vmax (0 µM) Vs. % Vmax (0.1µM), p = 0.9998, q = 0.1340, DF = 16; % Vmax (0 µM) Vs. % Vmax (0.33 µM), p = 0.6135, q = 1.374, DF = 16; % Vmax (0 µM) Vs. % Vmax (1.1 µM), p = 0.9565, q = 0.7451, DF = 16; % Vmax (0 µM) Vs. % Vmax (3.33 µM), p = 0.9995, q = 0.3264, DF = 16; % Vmax (0 µM) Vs. % Vmax (10 µM), p = 0.1066, q = 2.522, DF = 16; % Vmax (0 µM) Vs. % Vmax (20 µM), p = 0.0012, q = 4.778, DF = 16. For TH7126, % Vmax (0 µM) Vs. % Vmax (0.03µM), p > 0.9999, q = 0.0110, DF = 16; % Vmax (0 µM) Vs. % Vmax (0.1µM), p = 0.9752, q = 0.6637, DF = 16; % Vmax (0 µM) Vs. % Vmax (0.33 µM), p = 0.9465, q = 0.7789, DF = 16; % Vmax (0 µM) Vs. % Vmax (1.1 µM), p = 0.5413, q = 1.483, DF = 16; % Vmax (0 µM) Vs. % Vmax (3.33 µM), p = 0.2704, q = 1.977, DF = 16; % Vmax (0 µM) Vs. % Vmax (10 µM), p = 0.6716, q = 1.288, DF = 16; % Vmax (0 µM) Vs. % Vmax (20 µM), p = 0.6050, q = 1.387, DF = 16.

Figure S11. Raw Vmax and $K_{0.5}$ values of reactions from Fig. 4A-D, related to Figure 4.

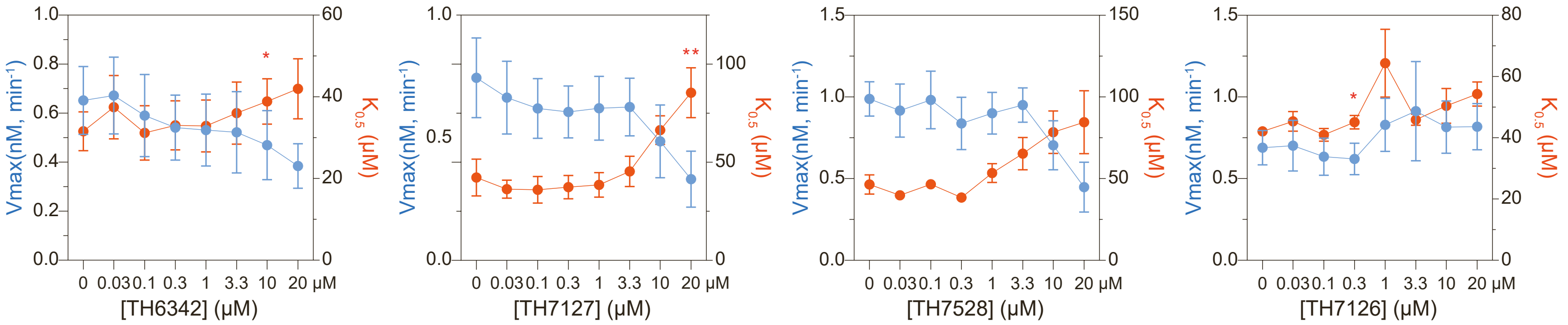

SAMHD1 inhibitors reduced the reaction Vmax, but increased $K_{0.5}$, as determined by fitting rate of reaction from Fig. 4A-D with an allosteric sigmoidal model (GraphPad Prism). Mean ± SEM of n = 3 independent experiments are shown. Raw Vmax and $K_{0.5}$ values are shown. Ordinary one-way ANOVA (Dunnett's multiple comparisons test) were performed between compound- versus DMSO-treated groups, asterisk signifies statistical significance (* for p ≤ 0.05, ** for p ≤ 0.01, *** for p ≤ 0.001, **** for p ≤ 0.0001). For $K_{0.5}$ comparisons – for TH6342, $K_{0.5}$ (0 μM) Vs. $K_{0.5}$ (0.03μM), p = 0.8425, q = 1.016, DF = 16; $K_{0.5}$ (0 μM) Vs. $K_{0.5}$ (0.1μM), p = 0.9977, q = 0.4111, DF = 16; $K_{0.5}$ (0 μM) Vs. $K_{0.5}$ (0.33 μM), p = 0.8343, q = 1.031, DF = 16; $K_{0.5}$ (0 μM) Vs. $K_{0.5}$ (1.1 μM), p = 0.1289, q = 2.416, DF = 16; $K_{0.5}$ (0 μM) Vs. $K_{0.5}$ (3.33 μM), p = 0.5769, q = 1.429, DF = 16; $K_{0.5}$ (0 μM) Vs. $K_{0.5}$ (10 μM), p = 0.0398, q = 3.042, DF = 16; $K_{0.5}$ (0 μM) Vs. $K_{0.5}$ (20 μM), p = 0.0653, q = 2.784, DF = 16. For TH7127, $K_{0.5}$ (0 μM) Vs. $K_{0.5}$ (0.03μM), p = 0.9929, q = 0.5254, DF = 16; $K_{0.5}$ (0 μM) Vs. $K_{0.5}$ (0.1μM), p = 0.9908, q = 0.5548, DF = 16; $K_{0.5}$ (0 μM) Vs. $K_{0.5}$ (0.33 μM), p = 0.9974, q = 0.4340, DF = 16; $K_{0.5}$ (0 μM) Vs. $K_{0.5}$ (1.1 μM), p = 0.9995, q = 0.3326, DF = 16; $K_{0.5}$ (0 μM) Vs. $K_{0.5}$ (3.33 μM), p = 0.9996, q = 0.2768, DF = 16; $K_{0.5}$ (0 μM) Vs. $K_{0.5}$ (10 μM), p = 0.2033, q = 2.153, DF = 16; $K_{0.5}$ (0 μM) Vs. $K_{0.5}$ (20 μM), p = 0.0079, q = 3.849, DF = 16. For TH7528, $K_{0.5}$ (0 μM) Vs. $K_{0.5}$ (0.03μM), p = 0.9943, q = 0.4982, DF = 16; $K_{0.5}$ (0 μM) Vs. $K_{0.5}$ (0.1μM), p > 0.9999, q = 0.0078, DF = 16; $K_{0.5}$ (0 μM) Vs. $K_{0.5}$ (0.33 μM), p = 0.9853, q = 0.6009, DF = 16; $K_{0.5}$ (0 μM) Vs. $K_{0.5}$ (1.1 μM), p = 0.9928, q = 0.5283, DF = 16; $K_{0.5}$ (0 μM) Vs. $K_{0.5}$ (3.33 μM), p = 0.5801, q = 1.424, DF = 16; $K_{0.5}$ (0 μM) Vs. $K_{0.5}$ (10 μM), p = 0.1319, q = 2.404, DF = 16; $K_{0.5}$ (0 μM) Vs. $K_{0.5}$ (20 μM), p = 0.0559, q = 2.866, DF = 16. For TH7126, $K_{0.5}$ (0 μM) Vs. $K_{0.5}$ (0.03μM), p = 0.9958, q = 0.4611, DF = 15; $K_{0.5}$ (0 μM) Vs. $K_{0.5}$ (0.1μM), p = 0.9998, q = 0.1618, DF = 15; $K_{0.5}$ (0 μM) Vs. $K_{0.5}$ (0.33 μM), p = 0.9976, q = 0.4201, DF = 15; $K_{0.5}$ (0 μM) Vs. $K_{0.5}$ (1.1 μM), p = 0.0370, q = 3.111, DF = 15; $K_{0.5}$ (0 μM) Vs. $K_{0.5}$ (3.33 μM), p = 0.9952, q = 0.4766, DF = 15; $K_{0.5}$ (0 μM) Vs. $K_{0.5}$ (10 μM), p = 0.7553, q = 1.170, DF = 15; $K_{0.5}$ (0 μM) Vs. $K_{0.5}$ (20 μM), p = 0.4119, q = 1.706, DF = 15. For Vmax comparisons – for TH6342, Vmax (0 μM) Vs. Vmax (0.03μM), p = 0.9999, q = 0.0978, DF = 16; Vmax (0 μM) Vs. Vmax (0.1μM), p = 0.9996, q = 0.3018, DF = 16; Vmax (0 μM) Vs. Vmax (0.33 μM), p = 0.9919, q = 5417, DF = 16; Vmax (0 μM) Vs. Vmax (1.1 μM), p = 0.9864, q = 0.5933, DF = 16; Vmax (0 μM) Vs. Vmax (3.33 μM), p = 0.9802, q = 0.6349, DF = 16; Vmax (0 μM) Vs. Vmax (10 μM), p = 0.9026, q = 0.8955, DF = 16; Vmax (0 μM) Vs. Vmax (20 μM), p = 0.6577, q = 1.308, DF = 16. For TH7127, Vmax (0 μM) Vs. Vmax (0.03μM), p = 0.9974, q = 0.4314, DF = 16; Vmax (0 μM) Vs. Vmax (0.1μM), p = 0.9745, q = 0.6672, DF = 16; Vmax (0 μM) Vs. Vmax (0.33 μM), p = 0.9573, q = 0.7420, DF = 16; Vmax (0 μM) Vs. Vmax (1.1 μM), p = 0.9756, q = 0.6611, DF = 16; Vmax (0 μM) Vs. Vmax (3.33 μM), p = 0.9799, q = 0.6366, DF = 16; Vmax (0 μM) Vs. Vmax (10 μM), p = 0.6067, q = 1.384, DF = 16; Vmax (0 μM) Vs. Vmax (20 μM), p = 0.1854, q = 2.208, DF = 16. For TH7528, Vmax (0 μM) Vs. Vmax (0.03μM), p = 0.9995, q = 0.3473, DF = 16; Vmax (0 μM) Vs. Vmax (0.1μM), p > 0.9999, q = 0.03254, DF = 16; Vmax (0 μM) Vs. Vmax (0.33 μM), p = 0.9565, q = 0.7329, DF = 16; Vmax (0 μM) Vs. Vmax (1.1 μM), p = 0.9975, q = 0.4289, DF = 16; Vmax (0 μM) Vs. Vmax (3.33 μM), p = 0.9997, q = 0.1809, DF = 16; Vmax (0 μM) Vs. Vmax (10 μM), p = 0.6086, q = 1.381, DF = 16; Vmax (0 μM) Vs. Vmax (20 μM), p = 0.0869, q = 2.633, DF = 16. For TH7126, Vmax (0 μM) Vs. Vmax (0.03μM), p > 0.9999, q = 0.05155, DF = 16; Vmax (0 μM) Vs. Vmax (0.1μM), p = 0.9997, q = 0.232, DF = 16; Vmax (0 μM) Vs. Vmax (0.33 μM), p = 0.9996, q = 0.2945, DF = 16; Vmax (0 μM) Vs. Vmax (1.1 μM), p = 0.9869, q = 0.5896, DF = 16; Vmax (0 μM) Vs. Vmax (3.33 μM), p = 0.8797, q = 0.9451, DF = 16; Vmax (0 μM) Vs. Vmax (10 μM), p = 0.9925, q = 0.5316, DF = 16; Vmax (0 μM) Vs. Vmax (20 μM), p = 0.9916, q = 0.5455, DF = 16.

Figure S12. The B4NPP direct SAMHD1 enzymatic assay is linear under the specified conditions, as well as for the duration of experiments, related to Figure 4.

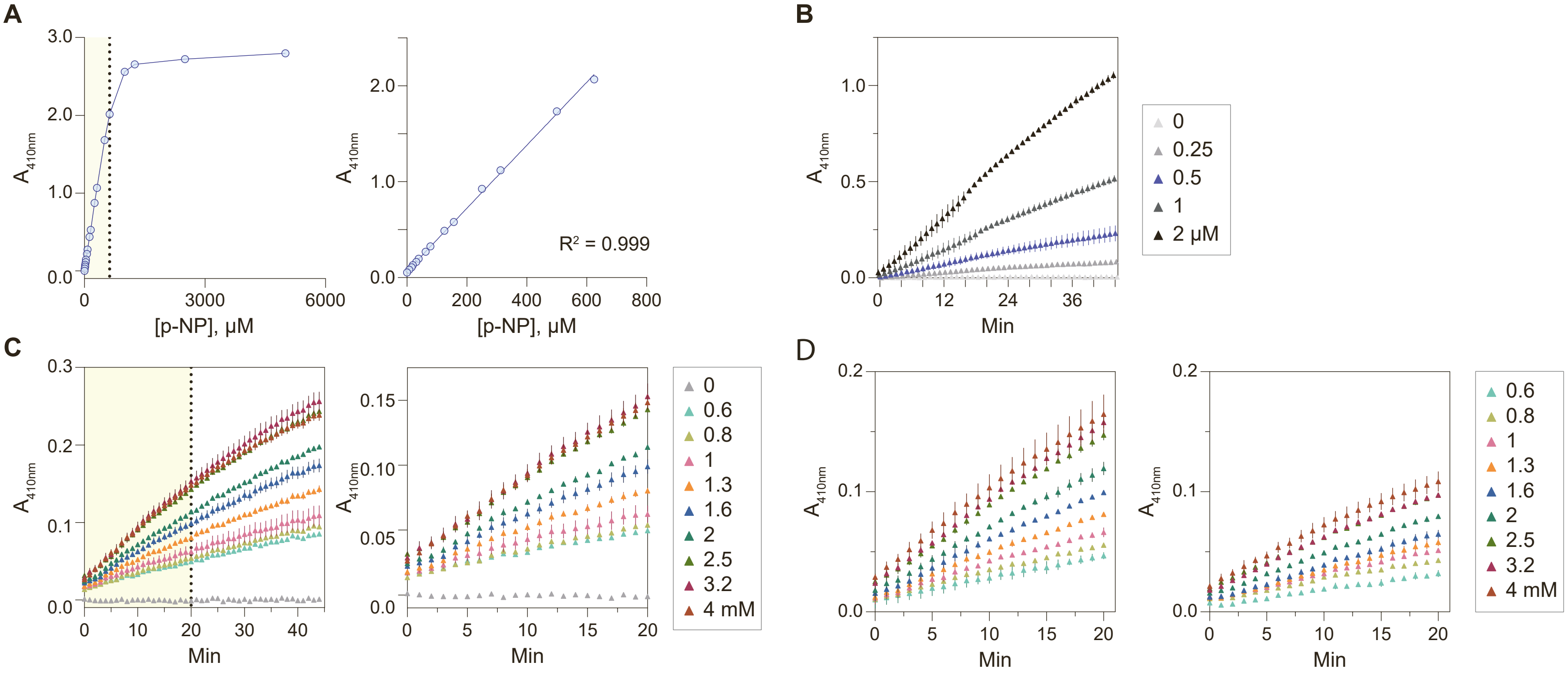

A. Establishment of linear range of detection of p-NP, product of B4NPP hydrolysis. Mean absorbance at 410 nm (A410nm) ± SEM of n = 2 independent experiments each performed in triplicate are shown. Left panel, A410nm up to 5 mM, with the linear range of detection highlighted. Right panel, determined linear range of detection, where the mean A410nm values were fitted through a simple linear regression model (GraphPad Prism). R2 value is shown. **B.** Titration of SAMHD1 concentrations. SAMHD1 of varying concentrations was incubated with 2 mM B4NPP, and A410nm was measured for up to 45 min. Mean A410nm ± SD of n = 2 independent experiments performed in triplicate or sextuplicate are shown. **C.** Titration of B4NPP concentrations. B4NPP at up to 4 mM was incubated with 0.5 μM recombinant SAMHD1 protein, and A410nm was measured for up to 45 min. Mean A410nm ± SEM of a representative experiments performed in triplicate are shown. Left panel, A410nm up to 45 min, with range of A410nm to determine V0 highlighted. Right panel, A410nm up to 20 min, signals used to determine V0 and subsequent kinetic study. **D.** Kinetic study of B4NPP hydrolysis by SAMHD1 under the specified conditions, in the presence of equivolume of DMSO (left panel) or TH7127 (right panel), supplementary to Fig. 4F. Mean A410nm ± SEM of n = 2 independent experiments performed in triplicate are shown, which were subsequently used to determine V0 and mode of inhibition of TH7127.

Figure S13. Supplemental information of target engagement assays on cellular SAMHD1 protein, related to Figure 6 and 7.

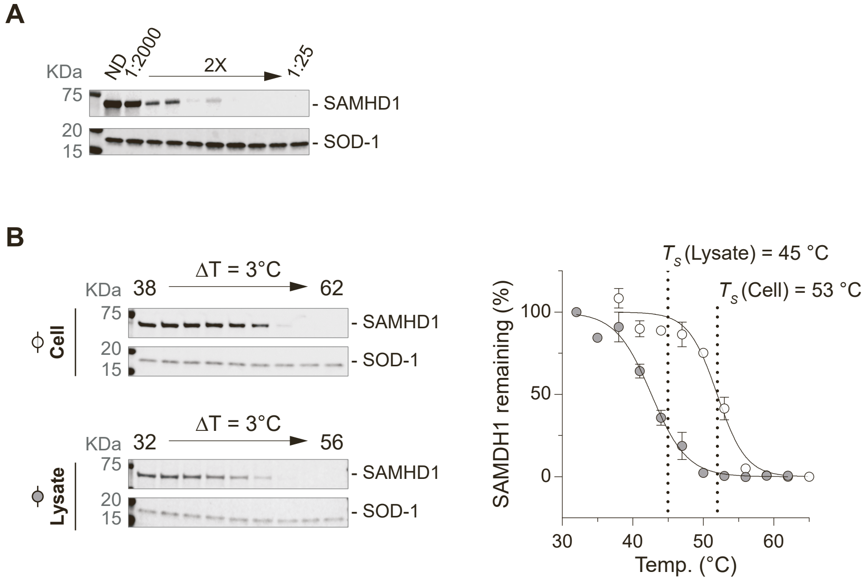

A. Optimisation of pronase concentration for SAMHD1 for DARTS experiments. Clarified THP-1 cell lysates were incubated with increasing concentrations of pronase, followed by Western blot analysis to determine the optimal pronase concentration for DARTS study of SAMHD1. **B.** Determination of screening temperatures in isothermal single-dose fingerprint CETSA experiments. Intact THP-1 cells or cell lysates were heated at increasing temperatures and were then analysed by Western blot for remaining soluble SAMHD1 protein. Left panels, representative Western blot images showing SAMHD1 and SOD-1 protein bands. Right panel, densitometry analysis of SAMHD1 signals, normalised to SOD-1 levels and then relative to samples heated at lowest temperatures. Mean relative SAMHD1 signals ± SEM of n = 2 independent experiments are shown. Melting curves are determined by curve-fitting mean relative SAMHD1 signals using a nonlinear regression model (Boltzmann sigmoidal, GraphPad Prism). Screening temperatures (T_s) are indicated.

Figure S14. The purity of recombinant SAMHD1 protein used in this study, related to Figure 1-4.

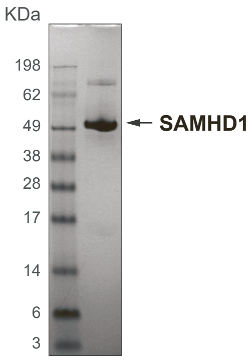

Approximately 4 µg of SAMHD1 protein was analysed using SDS-PAGE, followed by Coomassie blue staining. Protein concentration was calculated from UV absorbance at 280 nm using a theoretical extinction coefficient.

Table S1. HTS screening campaign information, related to Figure 1.

Category	Parameter	Description
Assay	Type of assay	in vitro target based
	Target	Deoxynucleoside triphosphate triphosphohydrolase, SAMHD1
	Primary Measurement	Absorbance at 630 nm using a coupled enzymatic assay to detect inorganic phosphate (Pi) using the malachite green assay
	Key reagents	Human recombinant SAMHD1; The coupled enzyme inorganic pyrophosphatase was produced in *E. coli* by Protein Science facility at Karolinska institutet.; dGTP (Sigma-Aldrich D4010); Stop reagent EDTA 7.9 mM; Malachite green detection reagents (Malachite Green, ammonium molybdate and Tween-20)
	Assay protocol	See supplementary information
	Additional comments	Protocol according to: Baykov, A. A., Evtushenko, O. A. & Avaeva, S. M. A malachite green procedure for orthophosphate determination and its use in alkaline phosphatase-based enzyme immunoassay. Anal. Biochem. 171, 266–270 (1988). Take care to ensure phosphate contamination in preparation of all reagents
Library	Library size	17656 compounds
	Library composition	The library consists of a chemically diverse collection of compounds containing both commercial (Enamine, TimTec, Maybridge and ChemDiv) and internal compounds (donation from Biovitrum). The library includes a small fraction of compounds with<br>known bioactivities, *e.g.* the Prestwick set, and a set of nucleosides from Barry Associates.
	Source	The screen was done based on plating of 10 mM DMSO solutions from Labcyte 384 LDV plates using an Echo 550
	Additional comments	See STAR method for further details on the compound library composition
Screen	Format	384-well format<br>Assay plate: 384-well PS plate, Nunc 242757
	Concentration(s) tested	Compound concentration at 5 µM, DMSO<br>concentration at 0.1%
	Plate controls	Positive control: buffer only representing fully inhibited SAMHD1 enzyme (16 on each plate) Negative control: uninhibited SAMHD1 enzyme (16 on each plate)
	Reagent/ compound dispensing system	Compound dispensing system: Echo 550 from<br>Labcyte<br>Reagent dispensing system: FlexDrop IV from PerkinElmer Multidrop from Thermo Scientific
	Detection instrument and software	Victor3 plate reader from PerkinElmer
	Assay validation/QC	Screen: Positive control: average absorbance 0.19, standard deviation 0.03. Negative<br>control: average absorbance 0.52, standard deviation 0.01. Average Z´ factor/plate: 0.75. QC also included monitoring of plate edge effects and distribution of the hits, with no corrections necessary
	Correction factors	Not applicable
	Normalization	Data are normalized to the positive (100% inhibition) and negative controls (0% inhibition) on each plate and are expressed as % inhibition
	Additional comments	The screen was performed at Chemical Biology Consortium Sweden at Karolinska Institutet, Sweden
Post-HTS analysis	Hit criteria	Hit threshold = Average "% inhibition" of all test samples (0,36%) + 3 times standard deviation of all test samples (3*7.60%) = 23.15%
	Hit rate	0.42%
	Additional assay(s)	Retesting of hits in 3 concentration hit confirmation experiment followed by a full concentration response experiments at 11 concentrations
	Confirmation of hit purity and structure	ID and purity analysis with LC-UV/MS detection
	Additional comments	

Table S3. Enzyme-coupled MG assay conditions, related to Figure 2.

Enzyme	Coupled enzyme	Substrate	Enzyme conc.
MTH1	PPase; 0.2U/ml	dGTP; 100 μM	4.8 nM
NUDT15	PPase; 0.2U/ml	dGTP; 100 μM	8 nM
NUDT5	BIP; 10U/ml	ADPR; 50 μM	6 nM
NUDT12	BIP; 10U/ml	β-NADH; 50 μM	20 nM
NUDT22	BIP; 10U/ml	UDP-glucose; 50 μM	30 nM
ITPase	PPase; 0.45U/ml	ITP; 50 μM	0.2 nM
dCTPase	PPase; 0.2U/ml	dCTP; 35 μM	35 nM
dUTPase	PPase; 0.4U/ml	dUTP; 12.5 μM	1.2 nM
